# Microarray analysis on germfree mice elucidates the primary target of a traditional Japanese medicine juzentaihoto: acceleration of IFN-α response via affecting the ISGF3-IRF7 signaling cascade

**DOI:** 10.1186/1471-2164-13-30

**Published:** 2012-01-18

**Authors:** Kaori Munakata, Kiyoe Takashima, Mitsue Nishiyama, Naoko Asano, Akihito Mase, Kyoji Hioki, Yasuyuki Ohnishi, Masahiro Yamamoto, Kenji Watanabe

**Affiliations:** 1 Center for Kampo Medicine, Keio University School of Medicine, Tokyo, Japan; 2 Tsumura Research Laboratories, Tsumura & Co., Ami, Ibaraki 300-1192, Japan; 3 Central Institute for Experimental Animals, 1430 Nogawa, Miyamae-ku, Kawasaki, Kanagawa 216-0001, Japan

## Abstract

**Background:**

The traditional Japanese medicine juzentaihoto (JTX) is a pharmaceutical grade multi-herbal medicine widely used for the prevention of cancer metastasis and infection in immuno-compromized patients in Japan. The effect of JTX has been supposed to be intimately affected by the immunological properties of host and enteric microflora. The influence of JTX on the gene expression profile in the large and small intestines was investigated by microarray analyses using mice of different strains with or without enteric microflora.

**Results:**

In all types of mice, including germfree (GF) animals, the genes most affected by two-week oral JTX treatment were the type 1 interferon (IFN)-related genes including Stat1, Isgf3g and Irf7, which play a critical role in the feedback loop of IFN-α production cascade. In IQI specific pathogen free (SPF) mice JTX increased the steady state level of the expression of IFN-related genes, but had the opposite effect in IQI GF and BALB/c SPF mice. Promoter analysis suggests that tandem repeated $IRFF (the promoter sequences for interferon regulatory factors) may be a primary target for JTX action. Pre-treatment of JTX accelerated the effects of an oral IFN "inducer" 2-amino-5-bromo-6-methyl-4-pyrimidinol (ABMP) (up-regulation of IFN-α production in IQI strain and down-regulation in BALB/c mice), which is in good accordance with the effect of JTX on gene expression of type 1 IFN-related genes.

**Conclusions:**

Microarray analysis revealed that the target of JTX might be the transcription machinery regulating the steady-state level of genes involved in the ISGF3-IRF7 cascade, whose effect is bi-directional in a strain- and microbiota-dependent manner.

## Background

In Japan, certain traditional herbal medicines (Kampo medicines), which comprise hot water extracts from a mixture of medicinal plants, have been widely used as ethical drugs and become integrated into the modern medical system [1-4]. These traditional medicines are manufactured under strict scientific quality control and are covered by public health insurance. A large amount of clinical and basic research on Kampo medicines has been performed, including more than 10 multicenter, placebo-controlled, double-blind studies.

We investigated the effects of Juzentaihoto (JTX) in this study. JTX is a well known Kampo medicine that comprises 10 different herbs; *Ginseng radix*, *Astragali radix*, *Angelicae radix*, *Rehmanniae radix*, *Atractylodis lanceae rhizoma*, *Cinnamomi cortex*, *Poria*, *Paeoniae radix*, *Ligustici rhizoma *and *Glycyrrhizae radix*. JTX has been used for centuries for the treatment of various kinds of disease or disorders such as anemia, rheumatoid arthritis, atopic dermatitis, chronic fatigue syndrome and ulcerative colitis [5-12]. Experimental studies show that JTX reduces the side effect of chemotherapy and radiotherapy [13-17], prevents various types of cancer and its metastasis [18-24], improves osteoporosis [25,26] as well as atopic dermatitis [1], and protects against Candida infection [19,27]. The active substances responsible for stimulation of hematopoietic stem cell growth [28,29], various immunostimulating activities [28,30-34], alleviation of side-effects by chemotherapeutic agents [32,35], and protection against Candida infection [36] have been identified.

In Kampo therapy, medical care is individualized with tailoring prescriptions depending on the patients' constitution, disease state and responsiveness to therapy [2,37,38]. Indeed, the therapeutic effects of Kampo-medicines have been known to vary markedly among individuals. Therefore, depending on the individual patient it is not unusual to use different Kampo drugs to treat the same disease, or alternatively, to use the same drug to treat apparently different diseases. Thus, the effect of Kampo medicines should be investigated by taking into consideration the possible dependence on the constitution, for example the immunological properties, of the individual patient.

Most Kampo medicines administered orally are known to have probiotic, prebiotic and antibiotic properties. For example, we have previously demonstrated that JTX alters the population of intestinal microflora, which affects the gene expression of heat shock proteins hsp70 and hsp110 in the gut and liver (Kampo affects microflora) [39]. Conversely, certain glycosides included in Kampo medicines are known to require metabolic conversion to a bioactive deglycosylated form by intestinal flora for expression of their pharmacological activity (Microflora affects Kampo) [2]. Finally, certain immunomodulating effects of Kampo medicines, including JTX, are known to be mediated by chemical components that are not thought to be absorbed [33,36,40]. The components of Kampo medicines may affect general immunity indirectly, presumably through interaction with the gut local immune system. Thus, the intestinal microflora is thought to play a critical role in eliciting the beneficial effects of Kampo medicines.

Because each Kampo medicine contains a vast number of ingredients, the biological effects are presumably mediated by multiple active components and their various target sites of action. Thus, a study involving any specific medicinal component or site of action is, in principle, inadequate. To clarify the action of such a complicated medicine requires an exhaustive approach such as transcriptome or proteome analysis. However, there have been very few studies that adopt an "omics" approach aimed at studying the pharmacology of Kampo medicines.

Here, we have attempted to resolve the above-mentioned problem by using the following approaches. 1) We investigated the effect of JTX on gene expression in the large intestines. As previously described, the effect of JTX extends over various organs and the entire body. However, the biological properties of JTX on the intestines may involve a broad spectrum of pharmacological effects. 2) We used GeneChip^® ^arrays to obtain a genome-wide profile of gene expression after JTX treatment. 3) We compared enteric flora-present specific pathogen-free (SPF) and enteric flora-absent Germ-free (GF) mice of the IQI strain to evaluate the influence of microflora on the action of JTX. 4) To determine the effect of the difference of immune properties the same experimental investigation was additionally performed on SPF Balb/c mice. Our investigation has identified a possible molecular pathway to explain the well-known effect of JTX in reducing the incidence of viral infections. Specifically, JTX enhances interferon-α production *via *elevation of steady-state expression level of interferon regulatory factors such as Stat1, Stat2, Isgf3γ and Irf7.

## Results

### Literature analysis of GeneChip data

**Figure 1:**
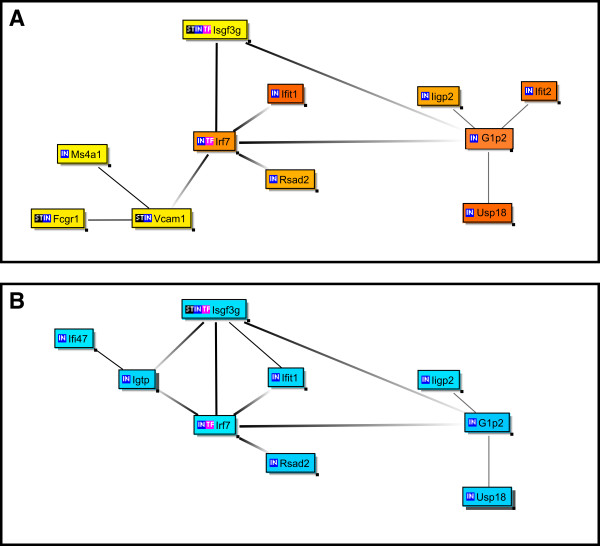
**Cluster Centered BiblioSphere for listed genes and co-cited transcription factors/genes**. The genes up-regulated in IQI SPF mouse large intestine (A) and the genes down-regulated in BALB/c mouse large intestine (B) are shown. A line indicates that the genes at both ends are co-cited in specific abstract(s) of PUBMED. A thick line with gray scale indicates that the gene at the dark end regulates expression of the genes at the lighter end i.e., transcription factor located upstream of the genes. A thick line in solid black indicates that the genes at both ends regulate the expression of each other. Genes with no co-citation have been omitted from the diagram. Orange, the genes whose fold change > 2; yellow, the genes whose fold change < 2; light blue, the genes whose fold change < 0.67.

Firstly, we investigated the effect of JTX on the steady state level of mRNA in the large intestines using IQI specific pathogen free (SPF) and Balb/c SPF mice (Experiment 1). When we applied the criteria described in *Materials and Methods *(p < 0.1, fold change > 1.5 or < 0.67), there were 27 and 16 annotated/identified genes whose expression level was increased by JTX treatment in IQI and Balb/c mice, respectively ("SPFLI-up" and "BALBLI-up" lists in Table 1 and Additional File 1, respectively). The numbers of down-regulated genes were 13 and 22 in IQI and Balb/c mice, respectively ("SPFLI-down" and "BALBLI-down" lists in Additional File 2 and Table 2 respectively). There were no common denominations between the up-regulated genes in IQI and those in Balb/c, and between the down-regulated genes in IQI and those in Balb/c. However, there was a striking commonality (8 genes, about 30% of listed genes) in the panels of genes that were up-regulated in IQI and genes down-regulated in Balb/c mice (bold letters in "SPFLI-up" and "BALBLI-down" lists, Tables 1 and 2). To elucidate the transcriptional co-regulation networks triggered by JTX, we used Genomatix' Bibliosphere software, which allows analyzing gene/gene, and gene/transcription factor relations from their co-citation in PubMed abstracts. The above gene lists were used as input to Bibliosphere. This approach allowed us to reveal relationships between the cluster of JTX-affected genes and other genes/transcription factors, which were co-cited with all or some of the genes from the input cluster, with the information as to whether these genes have certain promoter sequences. The results of this type of analysis, termed Cluster Centered BiblioSphere, are shown in Figure 1. Eleven (IQI) and 9 (Balb/c) genes are presented in Figure 1 and they have all been reported to be induced by type 1 interferon (other input genes were found to have no co-citation and therefore not presented in the figure) [41]. In particular, transcription factors Isgf3g and Irf7 are known to play a definitive role in the proximal sector in the signaling pathways mediating massive interferon-α production [42]. Accordingly, 6 of 8 genes that are common between the "SPFLI-up" and "BALBLI-down" lists contain promoter sequences for Irf7 or Isgf3g. Additionally, we analyzed the effect of JTX on the expression profile of the small intestine of IQI SPF mice. The profiles of the small intestine were completely different from, and had scarce commonality with, those of the large intestine of IQI SPF mice (Table 1 and Additional File 2, 3, 4).

**Table 1:** The upward effect of JTX on the gene expression in the large intestine in IQI SPF mice

SPFLI-up					
Probe Set ID	Gene Name	Gene Symbol	Entre ID	Fold Change	p-value
**93956_at**	**interferon-induced protein with tetratricopeptide repeats 3**	**Ifit3**	**15959**	**4.71**	**0.092**
**95024_at**	**ubiquitin specific peptidase 18**	**Usp18**	**24110**	**4.50**	**0.020**
**98822_at**	**ISG15 ubiquitin-like modifier**	**G1p2**	**53606**	**4.06**	**0.077**
**100981_at**	**interferon-induced protein with tetratricopeptide repeats 1**	**Ifit1**	**15957**	**3.84**	**0.033**
103066_at	thymidylate kinase family LPS-inducible member	Tyki	22169	3.72	0.012
103639_at	interferon-induced protein with tetratricopeptide repeats 2	Ifit2	15958	3.37	0.036
**104669_at**	**interferon regulatory factor 7**	**Irf7**	**54123**	**2.87**	**0.069**
98410_at	interferon inducible GTPase 2	Iigp2	54396	2.49	0.021
**104177_at**	**radical S-adenosyl methionine domain containing 2**	**Rsad2**	**58185**	**2.45**	**0.033**
**103335_at**	**lectin, galactose binding, soluble 9**	**Lgals9**	**16859**	**2.40**	**0.038**
93442_at	zinc finger protein 316	Zfp316	54201	1.97	0.065
161357_r_at	glutathione S-transferase, mu 2	Gstm2	14863	1.90	0.049
102867_at	TEA domain family member 4	Tead4	21679	1.82	0.009
100909_at	protease, serine, 8 (prostasin)	Prss8	76560	1.81	0.066
103657_i_at	metal response element binding transcription factor 2	Mtf2	17765	1.73	0.081
102879_s_at	Fc receptor, IgG, high affinity I	Fcgr1	14129	1.71	0.053
92560_g_at	vascular cell adhesion molecule 1	Vcam1	22329	1.69	0.036
95037_at	cyclin-dependent kinase 9 (CDC2-related kinase)	Cdk9	107951	1.69	0.086
104708_at	Transducin (beta)-like 1 X-linked	Tbl1x	21372	1.67	0.082
103884_at	ATP binding domain 1 family, member C	Atpbd1c	68080	1.66	0.061
99446_at	membrane-spanning 4-domains, subfamily A, member 1	Ms4a1	12482	1.63	0.083
**103634_at**	**interferon dependent positive acting transcription factor 3 gamma**	**Isgf3g**	**16391**	**1.58**	**0.068**
93464_at	A kinase (PRKA) anchor protein (yotiao) 9	Akap9	100986	1.54	0.088
92384_at	xeroderma pigmentosum, complementation group A	Xpa	22590	1.54	0.051
100472_at	enabled homolog (Drosophila)	Enah	13800	1.52	0.067
103080_at	SAM domain and HD domain, 1	Samhd1	56045	1.51	0.045
102808_at	Sodium channel, voltage-gated, type I, beta	Scn1b	20266	1.51	0.074

The genes whose change was > 1.50 fold, with p < 0.1 (n = 3, Welch's t test) were the listed sorted by fold-change. Unidentified 8 probe sets were omitted from the list. The genes appeared commonly in Tables 1 and 2 were in bold letters.

**Table 2:** The downward effect of JTX on the gene expression in the large intestine in Balb/c SPF mice

BALBLI-down					
Probe Set ID	Gene Name	Gene Symbol	Entre ID	Fold Change	p-value
160841_at	D site albumin promoter binding protein	Dbp	13170	0.27	0.029201
**104177_at**	**radical S-adenosyl methionine domain containing 2**	**Rsad2**	**58185**	**0.39**	**0.012171**
**95024_at**	**ubiquitin specific peptidase 18**	**Usp18**	**24110**	**0.41**	**0.016077**
160933_at	interferon gamma induced GTPase	Igtp	16145	0.46	0.052323
**100981_at**	**interferon-induced protein with tetratricopeptide repeats 1**	**Ifit1**	**15957**	**0.48**	**0.030177**
**98822_at**	**ISG15 ubiquitin-like modifier**	**G1p2**	**53606**	**0.49**	**0.053022**
103202_at	guanylate nucleotide binding protein 4	Gbp4	55932	0.52	0.018878
98410_at	interferon inducible GTPase 2	Iigp2	54396	0.55	0.008083
**93956_at**	**interferon-induced protein with tetratricopeptide repeats 3**	**Ifit3**	**15959**	**0.56**	**0.053353**
**103634_at**	**interferon dependent positive acting transcription factor 3 gamma**	**Isgf3g**	**16391**	**0.56**	**0.009681**
101561_at	metallothionein 2	Mt2	17750	0.59	0.001359
93573_at	metallothionein 1	Mt1	17748	0.61	0.00141
161964_r_at	Protein kinase C, zeta	Prkcz	18762	0.61	0.066851
**103335_at**	**lectin, galactose binding, soluble 9**	**Lgals9**	**16859**	**0.62**	**0.024207**
99076_at	Nuclear receptor subfamily 1, group D, member 2	Nr1d2	353187	0.63	0.049119
93085_at	proteosome (prosome, macropain) subunit, beta type 9	Psmb9	16912	0.63	0.00915
160208_at	splicing factor 3b, subunit 3	Sf3b3	101943	0.63	0.043591
104750_at	interferon gamma inducible protein 47	Ifi47	15953	0.64	0.053488
161287_f_at	MYB binding protein (P160) 1a	Mybbp1a	18432	0.64	0.014332
93178_at	neuronal guanine nucleotide exchange factor	Ngef	53972	0.65	0.017146
92315_at	schlafen 4	Slfn4	20558	0.65	0.053414
**104669_at**	**interferon regulatory factor 7**	**Irf7**	**54123**	**0.67**	**0.017792**

The genes whose change was < 0.67 fold, with p < 0.1 (n = 3, Welch's t test) were the listed sorted by fold-change. Unidentified 4 probe sets were omitted from the list "BALBLI-down", respectively. The genes appeared commonly in Tables 1 and 2 were in bold letters.

**Figure 2:**
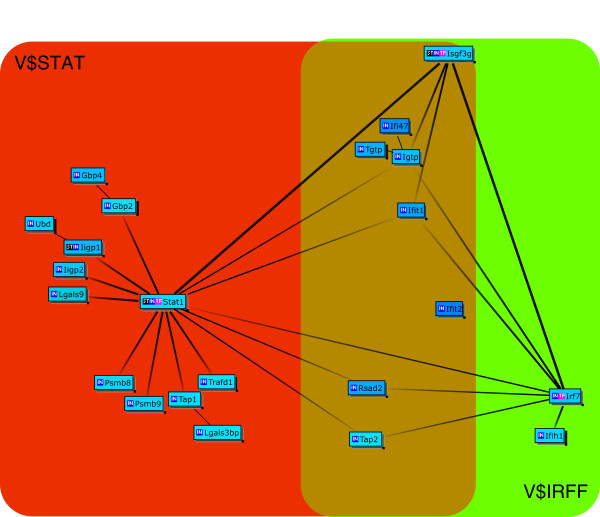
**Cluster Centered BiblioSphere for listed genes and co-cited transcription factors/genes**. The genes down-regulated in IQI GF mouse large intestine are presented. A line indicates that the genes at both ends are co-cited in specific abstract(s) of PUBMED. A thick line with gray scale gradation indicates that the gene at the dark end regulates expression of the genes at the lighter end i.e., transcription factor located upstream of the genes. A thick line in solid black indicates that the genes at both ends regulate the expression of each other. Genes with no co-citation have been omitted from the diagram. Light blue, genes whose fold change < 0.67. V$IRFF: genes having the promoter sequences for interferon regulatory factors, V$STAT: genes having the promoter sequences for signal transducers and activators of transcription genes. See website of Genomatix Inc http://www.genomatix.de/ for further information.

**Figure 3:**
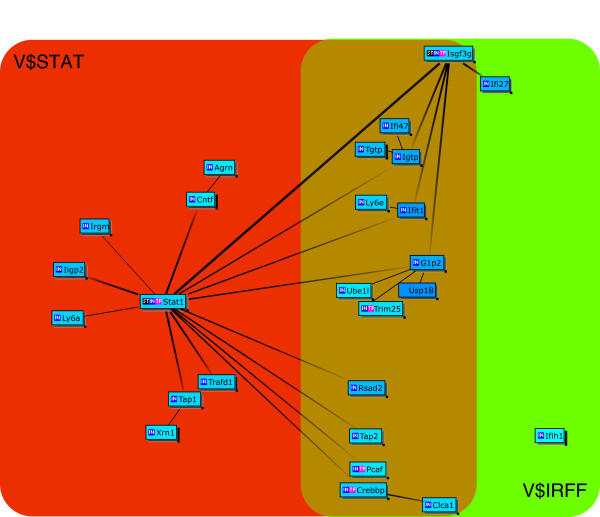
**Cluster Centered BiblioSphere for the listed genes and co-cited transcription factors/genes**. The genes down-regulated in IQI GF mouse small intestine are presented. A line indicates that the genes at both ends are co-cited in specific abstract(s) of PUBMED. A thick line with gray scale gradation indicates that the gene at the dark end regulates expression of the genes at the lighter end as the transcription factor. A thick line in solid black indicates that the genes at both ends regulate the expression of each other. The genes with no co-citation have been omitted from the diagram. Light blue, genes whose fold change < 0.67. V$IRFF: genes having the promoter sequences for interferon regulatory factors, V$STAT: genes having the promoter sequences for signal transducers and activators of transcription genes. See website of Genomatix Inc http://www.genomatix.de/ for further information.

Next we analyzed the GeneChip results of large and small intestines of IQI germ-free (GF) mice. In the large intestine, 13 genes have been listed as up-regulated ("GFLI-up" list in Additional File 5). Genes down-regulated by JTX treatment include 24 immunoglobulins. The "GFLI-down" list in Table 3 shows the other 31 annotated/identified genes. Seven (Irf7, Isgf3g, Ifit1, Ifit2, Iigp2, Rsad2 and Lgals9) of the 31 genes are common to those up-regulated in IQI SPF mice (i.e., "SPFLI-up" list in Table 1) and are known to be induced by type 1 interferons. The list of genes whose expression is significantly down-regulated in GF mice small intestines comprise 42 genes ("GFSI-down" list in Table 4). Unlike the results from SPF mice, the "GFSI-down" list (Table 4) shows a striking commonality with the "GFLI-down" list (15 of 31 genes, Table 3) and "SPFLI-up" list (9 of 27 genes, Table 1). The Bibliosphere analysis gave Cluster Centered Bibliosphere figures consisting of 23 out of 31 genes from "GFLI-down" list (Figure 2) and 26 out of 42 genes from "GFSI-down" list (Figure 3) that have been co-cited in PubMED abstracts. Figures 2 and 3 both include the two transcription factors, Isgf3g and Stat1, which are known to be located upstream of the Isgf3g-Irf7 signaling pathway. The contents of tables and supplementary tables are summarized in Additional File 6.

**Table 3:** The downward effect of JTX on the gene expression in the large intestine in IQI GF mice

GFLI-down					
Probe Set ID	Gene Name	Gene Symbol	Entre ID	Fold Change	p-value
**103842_at**	**DEAD (Asp-Glu-Ala-Asp) box polypeptide 3, Y-linked**	**Ddx3y**	**26900**	**0.02**	**0.004**
**103674_f_at**	**eukaryotic translation initiation factor 2, subunit 3, structural gene Y-linked**	**Eif2s3y**	**26908**	**0.07**	**0.008**
103639_at	interferon-induced protein with tetratricopeptide repeats 2	Ifit2	15958	0.09	0.015
**104750_at**	**interferon gamma inducible protein 47**	**Ifi47**	**15953**	**0.12**	**0.002**
**100981_at**	**interferon-induced protein with tetratricopeptide repeats 1**	**Ifit1**	**15957**	**0.18**	**0.021**
**104177_at**	**radical S-adenosyl methionine domain containing 2**	**Rsad2**	**58185**	**0.20**	**0.012**
**102906_at**	**T-cell specific GTPase**	**Tgtp**	**21822**	**0.24**	**0.034**
96764_at	interferon inducible GTPase 1	Iigp1	60440	0.27	0.001
**103202_at**	**guanylate nucleotide binding protein 4**	**Gbp4**	**55932**	**0.29**	**0.045**
**160933_at**	**interferon gamma induced GTPase**	**Igtp**	**16145**	**0.30**	**0.004**
**98410_at**	**interferon inducible GTPase 2**	**Iigp2**	**54396**	**0.36**	**0.014**
104669_at	interferon regulatory factor 7	Irf7	54123	0.38	0.034
103335_at	lectin, galactose binding, soluble 9	Lgals9	16859	0.38	0.039
100030_at	uridine phosphorylase 1	Upp1	22271	0.40	0.015
**103634_at**	**interferon dependent positive acting transcription factor 3 gamma**	**Isgf3g**	**16391**	**0.44**	**0.040**
92715_at	ubiquitin D	Ubd	24108	0.44	0.011
93085_at	proteosome (prosome, macropain) subunit, beta type 9 (large multifunctional peptidase 2)	Psmb9	16912	0.47	0.036
102791_at	proteosome (prosome, macropain) subunit, beta type 8 (large multifunctional peptidase 7)	Psmb8	16913	0.49	0.014
**103446_at**	**interferon induced with helicase C domain 1**	**Ifih1**	**71586**	**0.50**	**0.038**
**101465_at**	**Signal transducer and activator of transcription 1**	**Stat1**	**20846**	**0.51**	**0.026**
**103035_at**	**transporter 1, ATP-binding cassette, sub-family B (MDR/TAP)**	**Tap1**	**21354**	**0.54**	**0.035**
97950_at	xanthine dehydrogenase	Xdh	22436	0.54	0.010
104597_at	guanylate nucleotide binding protein 2	Gbp2	14469	0.56	0.025
97507_at	lectin, galactoside-binding, soluble, 3 binding protein	Lgals3bp	19039	0.60	0.047
102200_at	aquaporin 8	Aqp8	11833	0.60	0.019
**102873_at**	**transporter 2, ATP-binding cassette, sub-family B (MDR/TAP)**	**Tap2**	**21355**	**0.60**	**0.015**
**103254_at**	**TRAF type zinc finger domain containing 1**	**Trafd1**	**231712**	**0.60**	**0.011**
103892_r_at	elongation factor RNA polymerase II 2	Ell2	192657	0.62	0.039
99475_at	suppressor of cytokine signaling 2	Socs2	216233	0.66	0.024
92672_at	GNAS (guanine nucleotide binding protein, alpha stimulating) complex locus	Gnas	14683	0.67	0.010
98472_at	histocompatibility 2, T region locus 23///RIKEN cDNA C920025E04 gene	H2-T23	15040	0.67	0.007

The genes whose change was < 0.67 fold and with p < 0.1 (n = 3, Welch's t test) were the listed sorted by fold-change. Twenty four immunoglobulin genes and 14 unidentified probe sets were omitted from the lists. The genes appeared commonly in both Tables 3 and 4 were in bold letters. The genes appeared commonly in Tables 1 and 3 were underlined.

**Table 4:** The downward effect of JTX on the gene expression in the small intestine in IQI GF mice

GFSI-down					
Probe Set ID	Gene Name	Gene Symbol	Entre ID	Fold Change	p-value
**103842_at**	**DEAD (Asp-Glu-Ala-Asp) box polypeptide 3, Y-linked**	**Ddx3y**	**26900**	**0.01**	**0.006**
**103674_f_at**	**eukaryotic translation initiation factor 2, subunit 3, structural gene Y-linked**	**Eif2s3y**	**26908**	**0.03**	**0.022**
95024_at	ubiquitin specific peptidase 18	Usp18	24110	0.11	0.003
**100981_at**	**interferon-induced protein with tetratricopeptide repeats 1**	**Ifit1**	**15957**	**0.15**	**0.010**
93956_at	interferon-induced protein with tetratricopeptide repeats 3	Ifit3	15959	0.17	0.009
**160933_at**	**interferon gamma induced GTPase**	**Igtp**	**16145**	**0.19**	**0.000**
**98822_at**	**ISG15 ubiquitin-like modifier**	**G1p2**	**53606**	**0.20**	**0.035**
92718_at	interferon, alpha-inducible protein 27	Ifi27	76933	0.24	0.019
**104750_at**	**interferon gamma inducible protein 47**	**Ifi47**	**15953**	**0.25**	**0.009**
98030_at	tripartite motif protein 30	Trim30	20128	0.25	0.003
**104177_at**	**radical S-adenosyl methionine domain containing 2**	**Rsad2**	**58185**	**0.27**	**0.004**
**102906_at**	**T-cell specific GTPase**	**Tgtp**	**21822**	**0.29**	**0.000**
92472_f_at	schlafen 2	Slfn2	20556	0.31	0.004
**98410_at**	**interferon inducible GTPase 2**	**Iigp2**	**54396**	**0.32**	**0.007**
103066_at	thymidylate kinase family LPS-inducible member	Tyki	22169	0.36	0.024
97409_at	immunity-related GTPase family, M	Irgm	15944	0.36	0.021
**103446_at**	**interferon induced with helicase C domain 1**	**Ifih1**	**71586**	**0.39**	**0.010**
102699_at	myxovirus (influenza virus) resistance 2	Mx2	17858	0.42	0.012
93078_at	lymphocyte antigen 6 complex, locus A	Ly6a	110454	0.43	0.030
160668_at	opioid growth factor receptor	Ogfr	72075	0.44	0.006
**103254_at**	**TRAF type zinc finger domain containing 1**	**Trafd1**	**231712**	**0.44**	**0.043**
**103202_at**	**guanylate nucleotide binding protein 4**	**Gbp4**	**55932**	**0.44**	**0.013**
**101465_at**	**signal transducer and activator of transcription 1**	**Stat1**	**20846**	**0.44**	**0.021**
**103634_at**	**interferon dependent positive acting transcription factor 3 gamma**	**Isgf3g**	**16391**	**0.47**	**0.006**
**103035_at**	**transporter 1, ATP-binding cassette, sub-family B (MDR/TAP)**	**Tap1**	**21354**	**0.48**	**0.027**
103080_at	SAM domain and HD domain, 1	Samhd1	56045	0.50	0.016
103812_at	chloride channel calcium activated 1	Clca1	12722	0.50	0.019
160847_at	tRNA nucleotidyl transferase, CCA-adding, 1	Trnt1	70047	0.52	0.014
**102873_at**	**transporter 2, ATP-binding cassette, sub-family B (MDR/TAP)**	**Tap2**	**21355**	**0.57**	**0.037**
100475_at	tripartite motif protein 25	Trim25	217069	0.58	0.025
98283_at	5'-3' exoribonuclease 1	Xrn1	24127	0.61	0.005
96151_at	Molybdenum cofactor sulfurase	Mocos	68591	0.61	0.010
102812_i_at	ubiquitin-activating enzyme E1-domain containing 1	Ube1dc1	66663	0.62	0.003
94461_at	pre-B-cell colony-enhancing factor 1	Pbef1	59027	0.62	0.030
95886_g_at	CREB binding protein	Crebbp	12914	0.63	0.012
103673_at	complement component 2 (within H-2S)	C2	12263	0.63	0.013
97921_at	Agrin	Agrn	11603	0.63	0.043
102279_at	ubiquitin-activating enzyme E1-like	Ube1l	74153	0.64	0.013
104070_at	p300/CBP-associated factor	Pcaf	18519	0.64	0.002
96157_at	zinc finger protein 91	Zfp91	109910	0.65	0.009
94192_at	ganglioside-induced differentiation-associated-protein 10	Gdap10	14546	0.66	0.049
103025_at	Moloney leukemia virus 10	Mov10	17454	0.67	0.042

The genes whose change was < 0.67 fold and with p < 0.1 (n = 3, Welch's t test) were the listed sorted by fold-change. Eight immunoglobulin genes and 14 unidentified probe sets were omitted from the lists. The genes appeared commonly in both Tables 3 and 4 were in bold letters. The genes appeared commonly in Tables 1 and 4 were underlined.

**Figure 4:**
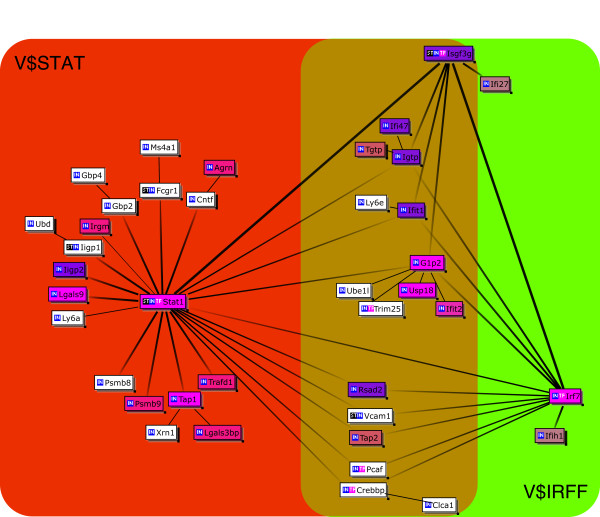
**A composite of Cluster Centered BiblioSphere of Figures 1, 2 and 3**. A line indicates that the genes at both ends are co-cited in specific abstract(s) of PUBMED. A thick line with gray scale gradation indicates that the gene at the dark end regulates the expression of genes at the lighter end i.e., as a transcription factor. A thick line in solid black indicates that the genes at both ends regulate the expression of each other. Genes with no co-citation have been omitted from the diagram. Violet, genes presented in all of 4 panels of Figures 1A, 1B, 2 and 3; salmon pink, genes presented in 3 of 4 panels; bright pink, genes presented in 2 of 4 panels; white, genes presented in 1 of 4 panels. V$IRFF: genes having the promoter sequences for interferon regulatory factors, V$STAT: genes having the promoter sequences for signal transducers and activators of transcription genes. See website of Genomatix Inc http://www.genomatix.de/ for further information.

Collectively, these data strongly suggest that JTX influences, at least in part, the signaling pathways of interferon-α production in the large intestines of different mice strains (IQI or Balb/c) and different microflora status (SPF or GF). These conclusions also apply to the small intestines of GF animals. The composite of Cluster Centered Bibliosphere figures of "BALB-down", "SPFLI-up", "GFLI-down" and "GFSI-down" figures are shown in Figure 4 suggesting almost all genes affected by JTX are under the regulation of either Stat1, Isgf3g or Irf7.

### Validation by quantitative real time RT-PCR

To confirm the results of GeneChip data, quantitative real time RT-PCR analysis has been performed on 26 genes, focusing on type 1 interferon-related genes. As shown in Table 5, many of the changes in interferon-related genes shown in GeneChip data have been confirmed by PCR. Messenger RNAs of IFN-β, IFN-α2 and IFN-α4, the end-products of signaling pathways of type 1 interferon production, were under the detection limit of real time PCR and unaffected by JTX treatment. These findings are in good agreement with the results of GeneChip analyses.

**Table 5:** Verification of the changes in gene expression by quantitative realtime RT-PCR

Gene Name	Gene Symbol	Gene ID	Effect of JTX (Fold Change)*		RT-PCR
			SPF	GF	primer/probe ID
**Interferon-reguratory factors**					
interferon regulatory factor 1 (IRF1)	Irf1	16362	1.18	1.00	Mm00515191_m1
interferon regulatory factor 3 (IRF3)	Irf3	54131	1.00	1.18	Mm00516779_m1
interferon regulatory factor 5 (IRF5)	Irf5	27056	**3.79**	1.46	Mm00496477_m1
interferon regulatory factor 6 (IRF6)	Irf6	54139	1.09	1.35	Mm00516797_m1
interferon regulatory factor 7 (IRF7)	Irf7	54123	**2.81**	**0.33**	Mm00516788_m1
interferon dependent positive acting transcription factor 3 gamma(IRF9)	Isgf3g	16391	**1.99**	0.73	Mm00492679_m1
interferon consensus sequence binding protein 1(IRF8)	Irf8	15900	1.00	1.40	Mm00492567_m1
**Interferon-inducible genes**					
interferon-induced protein with tetratricopeptide repeats 1	Ifit1	15957	**4.67**	0.30	Mm00515153_m1
interferon-induced protein with tetratricopeptide repeats 2	Ifit2	15958	**3.79**	**0.29**	Mm00492606_m1
glucocorticoid-attenuated response gene 49 (GARG-49/IRG2)	Ifit3	15959	**6.13**	**0.27**	Mm01704846_s1
guanylate nucleotide binding protein 2 (mGBP-2)	Gbp2	14469	1.10	1.03	Mm00494575_m1
ISG15 ubiquitin-like modifier	G1p2	53606	**6.00**	**0.23**	Mm01705338_s1
chemokine (C-X-C motif) ligand 10 (IP-10)	Cxcl10	15945	2.46	0.45	Mm00445235_m1
interferon inducible protein 1 (Ifi1)	Irgm	15944	**4.54**	0.94	Mm00492596_m1
lectin, galactose binding, soluble 9	Lgals9	16859	**2.53**	**0.58**	Mm00495295_m1
interferon gamma induced GTPase	Igtp	16145	**2.37**	**0.42**	Mm00497611_m1
lymphocyte antigen 6 complex, locus A (Ly6E.1, etc.)	Ly6a	110454	**2.25**	0.39	Mm00726565_s1
2'-5' oligoadenylate synthetase 1A	Oas1a	246730	**3.00**	NT	Mm00836412_m1
protein kinase, interferon inducible double stranded RNA dependent	Prkr	19106	**2.02**	NT	Mm00440966_m1
SAM domain and HD domain, 1 (Mgl1)	Samhd1	56045	1.25	1.62	Mm00490121_m1
**Type I interferon receptors**					
interferon (alpha and beta) receptor 1	Ifnar1	15975	1.04	**1.60**	Mm00439544_m1
interferon (alpha and beta) receptor 2	Ifnar2	15976	0.97	**1.44**	Mm00494916_m1
**Type 1 interferons**					
MuIFN-alpha-2 interferon-alpha-2 gene	Ifna2	15965	ND	NT	Mm00833961_s1
Interferon alpha family, gene 4	Ifna4	15967	ND	NT	Mm00833969_s1
**Stat family**					
Signal transducer and activator of transcription 1(Stat1)	Stat1	20846	**2.96**	NT	Mm00439518_m1
Signal transducer and activator of transcription 2(Stat2)	Stat2	20847	**4.24**	NT	Mm00490880_m1

* Fold changes statistically significant (P < 0.05) are indicted in bold letter. NT: not tested.

### Promoter analysis of GeneChip data

**Figure 5:**
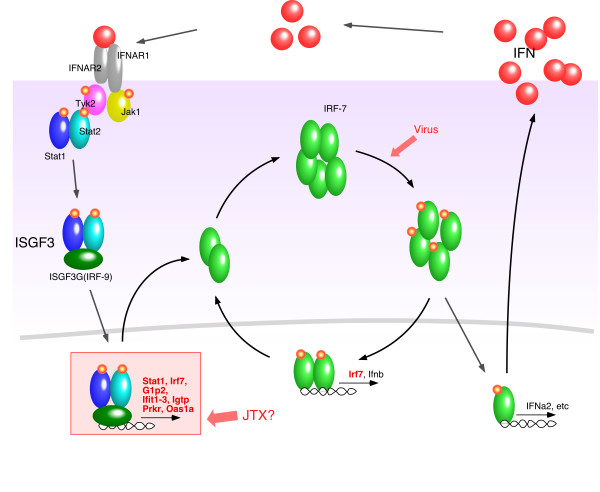
**Diagram of the signaling cascade of IFN-α production and a possible target for the action of JTX**.

**Figure 6:**
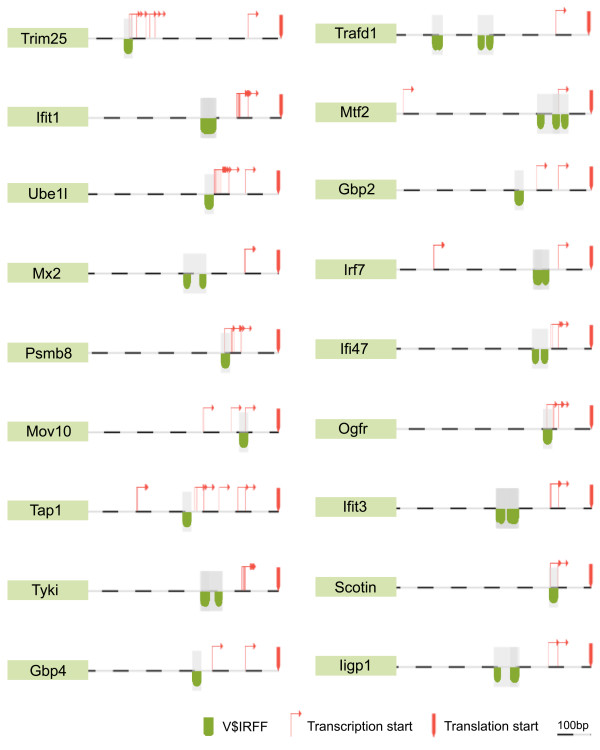
**The location of V$IRFF-V$IRFF cassettes in JTX-affected genes**.

Genes, whose expression profiles changed according to the GeneChip and PCR data analysis, were further investigated. Several of these genes were found to act on the signaling pathway map of type 1 interferon production. The effect of JTX appeared to focus on the neighborhood of the ISGF3 signaling pathway (Figure 5). To investigate the transcription regulation working behind the change of gene transcription, we performed promoter sequence analysis using Genomatix' ElDorado/Gene2Promoter/FrameWorker system to find transcription factor motifs in similarly regulated genes. Co-regulation of mammalian genes usually depends on sets of transcription factors rather than an individual factor alone. Regulatory sequence elements are often organized into defined frameworks of motifs of two or more transcription factor binding sites and clusters of such motifs. The FrameWorker software combined with the genome annotation database/interface ElDorado/Gene2Promoter makes it possible to retrieve common frameworks in the promoter region of the input genes. Twelve genes that were commonly identified in more than 3 lists of "BALB-down", "SPFLI-up", "GFLI-down" and "GFSI-down" (see Figure 5) were analyzed. Twenty models have been found as the operable modules consisting of two transcription factors. Four models with the lowest FW-scores (V$IRFF-V$IRFF, 1.43974e-05; V$IRFF-V$MYT1, 1.73886e-05; V$CREB-V$GCMF, 1.7771e-05; V$ETSF-V$DEAF, 5.56327e-05) were subjected to further examination. The appearance ratio of each model in the genes affected by JTX treatment and in the all annotated mouse promoters are shown in Table 6. The average and SD of the ratio of four cases (SPFLI-up, BALB-down, GFLI-down, and GFSI-down) was statistically compared and significant differences were found for V$IRFF-V$IRFF, V$IRFF-V$MYT1 and V$CREB-V$GCMF. The location of V$IRFF-V$IRFF in JTX-affected genes is illustrated in Figure 6.

**Table 6:** Putative promoter modules affected by JTX treatment

module	# module matches (# sequences)					Appearance ratio (# matches/# sequences)		Significance
		
	SPFLI-up (26)	BALB -down (26)	GFLI -down (45)	GFSI down (55)	All promoters (51460)	SPFLI-up/BALB-down/GFLI-down/GFSI-down*	All annotated promoters	
V$IRFF-V$IRFF	31	33	55	67	19374	1.225 ± 0.032	0.376	< 0.0001
V$IRFF-V$MYT1	14	12	24	30	13423	0.520 ± 0.039	0.261	0.0009
V$CREB-V$GCMF	4	6	10	14	4675	0.215 ± 0.043	0.091	0.0104
V$ETSF-V$DEAF	3	8	17	17	6540	0.277 ± 0.113	0.127	0.0706

* The values represent the mean ± S.D. of the appearance ration of these 4 groups.

Definition of promoter modules**

V$IRFF: interferon regulatory fatctors,

V$MYT1: MYT1 C2HC zinc finger protein,

V$CREB: cAMP-responsive element binding proteins,

V$GCMF: Chorion-specific transcription factors with a GCM DNA binding domain,

V$ETSF: Ets1 factors,

V$DEAF: Homolog to deformed epidermal autoregulatory factor-1 from D. melanogaster

**See Website of Genomatix Inc http://www.genomatix.de/ for further information.

### JTX enhanced the ABMP-induced increase of IFN-alpha production in the large intestine of IQI SPF mice

To investigate whether the difference of basal expression levels of the IFN-related genes results in the altered production of IFN-α, we examined the effect of IFN-inducer on production of IFN-α protein in the large intestines (Experiment 2). Firstly, we compared the effect of three oral IFN-α inducers, 2-Amino-5-bromo-6-methyl-4-pyrimidinol (ABMP), tilorone analog R11567DA and imiquimod. Oral administration of 30 mg/kg body weight of Imiquimod, a TLR7 agonist, rapidly (2 hr) increased the IFN-α protein level of serum but not that of the intestines. Oral administration of 100 mg/kg body weight of R11567DA increased the level of IFN-α in both the serum and intestines within 4 hr. The increase in IFN-α level in the intestines induced by ABMP took place over a prolonged period of time (30 hr) and was relatively modest. However, ABMP did not induce IFN-α production in the serum. Therefore ABMP was thought to be suitable for evaluation of the net increase of intestinal IFN-α production. Hence, in the following experiments we chose to use ABMP. The effects of ABMP at the doses of 250, 500, 1000 mg/kg body weight have been compared and 250 mg/kg was determined as the suitable dosage (data not shown).

**Figure 7:**
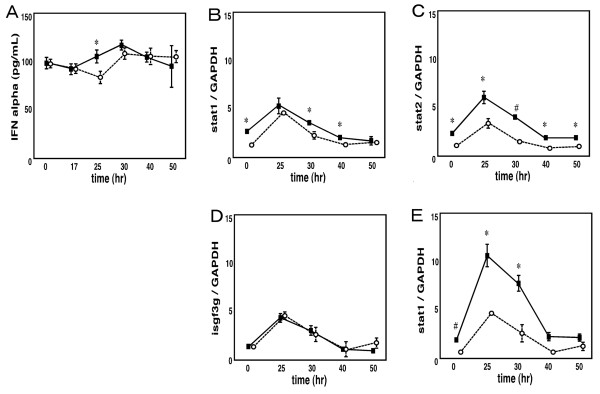
**ABMP-induced change in IFN-α production in the large intestine of IQI SPF mice after administration of either water (open circle) or JTX (closed square)**. a: Amount of IFN-α protein. The release of IFN-α began earlier in JTX-treated mice than in the control mice. b-e: Gene expression of Stat1, Stat2, Isgf3g, which compose an ISGF3 complex, and Irf7. The peak amount of all mRNA expression of Stat2 and Irf7 increased in JTX-treated mice. *p < 0.05. JTX-treated *vs*. control.

As shown in Figure 7, in the absence of stimulation by ABMP, the level of IFN-α protein in the large intestine was unaffected by a two week administration of JTX in IQI mice. However, in accordance with the GeneChip data, expression levels of Stat1, Stat2 and Irf7 were increased. When ABMP was administered in JTX-treated mice, maximum concentration of IFN-α protein induced by ABMP stimulation showed no change. However, IFN-α release peaked much earlier in mice given ABMP compared with the control mice. Furthermore, JTX treatment increased the peak level of Stat1, Stat2 and Irf7 mRNAs.

**Figure 8:**
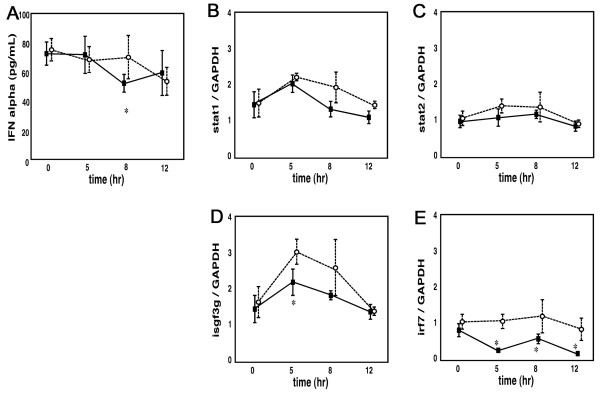
**ABMP-induced change in IFN-α production in the large intestine of Balb/c mice treated with water (open circle) or JTX (closed square)**. a: Amount of IFN-α protein. The decrease of IFN-α began earlier in JTX-treated mice than in the control mice. b-e: Gene expression of Stat1, Stat2, Isgf3g, and Irf7. The level of Isgf3g and Irf7 mRNAs was lower than the control at JTX-treated mice.

Similar experiments using the same protocol were performed on another strain of Balb/c mice (Figure 8). IFN-α protein levels in the large intestine were unaffected by a two week administration of JTX alone. However, in accordance with GeneChip data, the steady-state levels of Stat1, Stat2 and Isgf3g decreased in JTX-treated mice. To our surprise, administration of an "oral IFN inducer" ABMP resulted in a significant decrease in IFN-α release after 12 hr. The mRNA expression of Stat1, Stat2 and Isgf3g was induced by ABMP, but the Irf7 mRNA level showed no change. JTX treatment did not affect the basal level of IFN-α release, although the decrease in the level of IFN-α was evident after only 8 hr in JTX-treated mice i.e., earlier than the control. JTX decreased the level of Isgf3g and Irf7 mRNAs compared to the control.

## Discussion

Application of comparative functional genomics to pharmacology may make it possible to classify the biological effects of a certain drug into common universal effects and host-specific and/or anecdotal effects. Such a strategy is particularly useful for investigating the pharmacology of drugs with composite properties, such as combination drugs, phytomedicines and traditional medicines that use relatively crude ingredients. This is because these medicines generally have multiple active components through which they target several different molecules to exert their pharmacological efficacy. In the present study, we performed transcriptome analyses to profile the effect of a Kampo (Japanese traditional herbal) medicine, JTX. Different mice strains, whose immunological properties have been reported to be distinctive, were used. Furthermore, to classify the microflora-dependent and -independent effect of JTX, we used GF and SPF mice of the same IQI strain. Our results have identified genes commonly affected by JTX treatment across the different strains/status of mice, which presumably represent the principal biological effects of this Kampo medicine. However, the effects by JTX on expression of these genes were directionally different among the strains/status (i.e., down-regulated in BALB/c and IQI GF mice, up-regulated in IQI SPF mice). Our data suggests the following conclusions: 1) JTX has common target molecule(s), which may affect the expression of type-1 IFN-related genes, such as Ifit1, Stat1 and Irf7; 2) the active principles of JTX responsible for this phenomenon do not require the metabolic conversion by intestinal microflora because the same genes were affected in JTX-treated GF mice; 3) the functions of these molecules may be quite different depending on the host strain/status. This is a plausible explanation because the genes commonly affected by JTX are the immune-related genes whose outcomes are known to be drastically different (sometimes opposite) depending on the strains [43] and the presence/absence of intestinal microflora [44]. Furthermore, this finding appears to be in good accordance with the concept that Kampo medicines should be administered according to the "Sho". "Sho" is a principle of Kampo medicine that recognizes the physical constitution, immunological predisposition and responsiveness to the drugs can vary between individual patients [7]. The precise interpretation of "Sho" in terms of modern medical science is yet to be fully defined. Nonetheless, "Sho" is thought to be intimately related to the patient's immunological responsiveness and the state of intestinal microflora. Similarly, we have demonstrated shosaikoto, another Kampo medicine, to have different, possibly even opposite, effects on lung immunity in hosts with different genetic backgrounds [45,46].

Interestingly, the genes affected by JTX were dissimilar between the small and large intestine of SPF mice while the genes affected by JTX have a lot of commonality between these tissues in GF mice. As described in the RESULTS section, the "GFSI-down" list (comprising 42 genes) share 15 genes with the "GFLI-down" list (comprising 31 genes) and 9 genes with the "SPFLI-up" list (comprising 27 genes), respectively, while both the "SPFSI-up" list (Additional File 3) and "SPFSI-down" list (Additional File 4) share no genes with any other "up/down" lists of "GFSI" (Additional File 7 and Table 4), "GFLI" (Additional File 5 and Table 3) and even "SPFLI" (Table 1 and Additional File 2). Thus, our results suggest that the small intestine of SPF mice differentiate *via *a distinct process. The "SPFSI" list includes many metabolic enzymes. This suggests an extensive and large scale alteration of the metabolic system in the SPF small intestine. It has been reported that inoculation of bacteria into GF mice resulted in profound changes in xenobiotic metabolism [47]. An extensive alteration of small intestinal brush border enzymes between GF and ex-GF piglets has also been noted [48]. Furthermore, a recent paper has suggested a three-way interaction between the immune system, the intestinal metabolism and the microbiota [49,50].

In this study, in both IQI and BALB/c strains, a similar panel of IFN-related genes was affected by JTX treatment. The panels include genes involved in signal pathway of IFN-αinduction/production, such as Stat1, Isgf3g and Irf7, and genes known as "IFN-stimulated genes", which are closely related to IFN-αinduction/production, such as Ifit1, G1p2 (also known as ISG15), Igtp, Ifi47, Tgtp, Lgals9 and Rsad2 [41]. These findings have addressed the possibility that JTX may exert its pharmacological effect *via *modulating the signaling pathway for IFN production. In particular, Irf7, is a rate limiting transcription factor located in the center of a self-amplifying positive feedback loop of massive IFN-α production [42]. Microbial infection induces phosphorylation of IRF7 protein, which is then transported into nuclei to strongly induce the expression of IFN-αs and Irf7 itself. In the presence of a kinase activated by infectious agents, newly synthesized IRF7 continues to activate the loop resulting in a massive production of IFN-αs. The inducibility of IFN-α production in various tissues/cells and their steady state level of IRF7 proteins are known to be closely correlated [51]. Isgf3g (Irf9) and Stat1 are two components of ISGF3, a signaling complex that transduces the signal from receptors of IFN-α/β and induces the expression of IFN-α-related molecules, including G1p2, Stat1 and Irf7 [52]. By contrast, the expression of other interferon regulatory factors, such as Irf1, Irf2 and Irf8, did not change. Indeed, the end-products of the pathway, such as the IFN-αs, α2 and α4, were undetectable. These data suggest that JTX treatment by itself does not affect IFN-α expression directly. However, the steady-state expression level of genes responsible for induction of IFN-α synthesis are altered by JTX. Thus, once the stimulatory signals are triggered, JTX-treated mice produce altered levels of IFN-α protein. This conclusion has been confirmed by the experiments using an oral interferon inducer, ABMP. Although pretreatment by JTX did not change the maximum level of IFN-α, the drug did accelerate ABMP-induced changes in the production of IFN-α protein.

In the early phase of infectious diseases, the prompt supply of amounts of IFN-α protein effectively eliminates viruses and prevents the dissemination of the infectious agents into the whole body. Intriguingly, another Kampo medicine, hochuekkito (HET) has been reported to reduce the lethality due to influenza infection not by increasing the total (or peak) amount of IFN-α but by accelerating the onset of IFN-α release in the lung [53]. It is not known whether the acceleration of IFN-α response by HET is mediated *via *transcriptional up-regulation of the genes involved in IFN-α production, i.e., Stat1, Isgf3 or Irf7. It is also unclear whether JTX accelerates infection-triggered IFN-α production or indeed whether the JTX-induced preventive effects against infectious diseases are related to its acceleration of IFN synthesis signaling. In spite of the apparent similarities between HET and JTX, beneficial effects of these drugs have been suggested to have different characteristics and responder patients, both clinically and experimentally [13,54]. The comparative investigation of effects of both drugs on viral infection and IFN-α production/degradation signaling pathways is now in progress in our laboratory.

JTX by itself does not increase nor decrease IFN-α production. Furthermore, the results of JTX treatment were different among strains/enteric flora status. Steady-state levels of IFN-related genes were up-regulated in IQI SPF mice but down-regulated in IQI GF and BALB/c mice. A stimulation by ABMP also exerted opposite effects on IFN-α levels between IQI SPF and BALB/c mice suggesting an additional, strain-specific regulatory pathway(s) may exist upstream of the ISGF3 complex. It is yet to be determined whether the down-regulation of IFN-α responses by JTX is involved in the clinical effects of JTX. As well as playing a critical role in defense against viral infections, type I IFN also has a pivotal role at the interface between innate and adaptive immunity. In spite of the marked efficacy of recombinant IFN-α therapy, the cytokine has been reported to be involved in the development or exacerbation of numerous autoimmune phenomena, including a variety of neuropathy syndromes, neuromuscular junction disorders and myopathies [55-57]. JTX displays various immunomodulating activities [28,29,31,33,34,58-64]. These effects should be investigated in the scope of a complex interplay of many genetic and environmental factors that influence the balance between normal and aberrant immune responsiveness. The elucidation of mechanism(s) of action by which JTX (and, presumably, ABMP) exerts its influence on IFN-α synthesis/degradation may lead to the elucidation of a novel regulatory pathway of IFN-α regulation. Furthermore, the identification of the target cell population of JTX may contribute to the discovery of a novel type of IFN-α producing cells. We [41] and other researchers [65] have found that colonic IFN-α producing cells may be distinct from authentic plasmacytoid dendritic cells, the well-known IFN-α producer.

## Conclusion

In the present study, by applying microarray analysis to different strains/status of mice, we have tried to elucidate an essential component of the pharmacological effect of JTX. Our results reveal that JTX modulates the potency of IFN-α production by affecting the genes related to the ISGF3 complex-IRF7 loop, rate-limiting transcription factors involved in the IFN-α production signaling pathway. We are currently attempting to identify the active ingredients of JTX and elucidate their respective mechanisms of action.

## Methods

### Animals

Male IQI/Jic mice from CIEA (Kanagawa, Japan) and BALB/c mice from Charles River Japan (Yokohama, Japan Inc.) were kept under specific pathogen-free (SPF) conditions. Germ-free (GF) mice were housed in a Trexler-type flexible film isolator in a standard germ**-**free state and screened on a weekly basis for germ-free status by sampling feces sterilely and culturing on MRS-agar plates under aerobic and anaerobic conditions.

All mice used in this study were 7-9 weeks old. Mice were housed in an air-conditioned room (temperature 24 ± 1°C) with a controlled light/dark cycle (light on between 6:30 AM and 7:00 PM). Food and water were available *ad libitum*. The mice were randomly divided into two groups; three to six mice were included in each group. One group was orally treated with JTX solution (1.0 g/kg body weight) and the other was treated with an equal volume of water as control. JTX was administrated using a stainless steel gastric tube once a day for 14 days. All animal procedures were approved by the institution's ethical committee for care and use of laboratory animals in research.

### Drugs

JTX, consisting of spray-dried hot water extracts of a mixture of ten medicinal plants, was obtained from Tsumura & Co. (Tokyo, Japan). The mixture consisted of *Astragali radix *(3.0 g), *Cinnamomi cortex *(3.0 g), *Rehmanniae radix *(3.0 g), *Paeoniae radix *(3.0 g), *Ligustici Rhizoma *(3.0 g), *Cnidii rhizoma*, *Atractylodis lanceae rhizome *(3.0 g), *Angelicae radix *(3.0 g), *Ginseng radix *(3.0 g), *Poria *(3.0 g) and *Glycyrrhizae radix *(3.0 g). The origins and species of herbs, the contents of characteristic and active ingredients and other pharmaceutical-grade qualities of JTX have been strictly controlled as the ethical drug approved by the Ministry of Health, Welfare and Labor of Japan. ABMP (2-Amino-5-bromo-6-methyl-4-pyrimidinol), an oral interferon inducer [66-68], was obtained from ACROS ORGANICS (Fair Lawn, NJ).

### Experiment 1

GF and SPF male IQI mice and SPF male BALB/c mice (7 weeks old, n = 6 for each group) were orally treated with JTX solution (0.1 g/ml/100 g body weight) or water daily for 14 days. Whole portions of the small and large intestine were homogenized and an aliquot of each homogenate was subjected to total RNA extraction. Labeled cRNA prepared from 3 mice per group was hybridized to the GeneChip Murine Genome U74A V.2 (Affymetrix). Real time RT-PCR was used to confirm the results of the GeneChip analysis using total RNA prepared from the other 3 mice (per group).

### Experiment 2

SPF male IQI and BALB/c mice (7 weeks old) were orally treated with JTX solution (0.1 g/ml/10 g body weight) or water daily for 2 weeks. Then an oral IFN inducer, ABMP (2-Amino-5-bromo-6-methyl-4-pyrimidinol) was administered once. ABMP was dissolved in water to become 250 mg/kg and vortexed. At various times the large intestines were taken, rinsed in phosphate-buffered saline (PBS), and cut longitudinally. One half was used for gene expression analysis and the other half for IFN-α ELISA. Both samples were flash-frozen in liquid nitrogen and stored at -80°C until use.

### Obtaining of tissue samples

ABMP was administered and the large intestines were obtained at various times. The tissue was rinsed in phosphate-buffered saline (PBS) and cut longitudinally. One half was used for gene expression analysis and the other half for IFN-α ELISA. Both samples were flash-frozen in liquid nitrogen and stored at -80°C until use.

### RNA extraction from mice tissue

Mice were sacrificed and the large intestine taken for preparation of total RNA. Each frozen sample was homogenized in a 1 ml/0.1 g tissue of TRI REAGENT (Sigma-Aldrich Japan, Tokyo, Japan) with a POLYTRON tissue homogenizer (Kinematica, Littau-Lucerne, Switzerland) and incubated for 10 minutes at RT. Chloroform (0.2 ml/1 ml TRI REAGENT) was added to the samples and the suspensions were centrifuged at 13,200×*g *for 15 min at 4°C. The water phase was transferred to a new tube and the RNA prepared with a conventional isopropanol/ethanol precipitate technique. To check the quality and quantity of RNA, UV absorbance at 260 nm was determined and the samples were also electrophoresed and visualized under UV illumination after staining with ethidium bromide.

### Microarray Analysis

Total RNA was extracted from mice (n = 3 per each group) using TRIzol (Life Technologies, Rockville, TX) and re-purified by RNeasy spin columns (Qiagen, Valencia, CA) according to the manufacturer's instructions. All samples were monitored using an Agilent Bioanalyzer (Agilent Biotechnologies, Boeblingen, Germany) and consistently demonstrated high-quality RNA (28S/18S ratio, ~2). The labeled cRNA prepared by *in vitro *transcription (Enzo Biochem, New York, NY) was fragmented, hybridized to a MG-U74Av2 array (Affymetrix, Santa Clara, CA) using an Affymetrix fluidics station, and scanned with an Affymetrix scanner, according to the Affymetrix protocol. The array contains 12488 probe sets. Data were deposited to Gene Expression Omnibus [69] and available under the series IDs: GSE8006, GSE32083 and GSE32084. Data were analyzed using the Affymetrix Microarray Suite (MAS) v.5.0 with all of the parameters set at default values (a global normalization was applied). The probe sets that had 2 or 3 absent A MAS detection calls per group (3 samples) in all groups were excluded. Therefore, genes that had more than 2 present calls in any one of the groups were included in the analysis. Further, probe sets with signal intensities less than 50 were omitted because preliminary evaluation by RT-PCR revealed poor reproducibility for genes with lower signal intensities. Statistical analyses were performed by Welch's t-test. This procedure have been used in a series of our project including our previous papers [39,41] and we have found that the criteria of p < 0.1 is usable for obtaining data which is worth additional validation experiments providing we use the same protocols and that the animals are sourced from the same provider (Central Institute for Experimental Animals, Kanagawa, Japan). Some portions of the microarray data are represented as heat maps in Additional File 8.

### Promoter analysis

Analysis of the sequences for transcription factor binding sites was conducted with the program MatInspector Professional (Genomatix Software, Munich, Germany) based on the MatInspector program using the selected matrix library (vertebrate section) and optimized thresholds. The promoter modules generated were compared with the database using ElDorado software.

### Reverse Transcription

Total RNA was extracted in a modified acid guanidium thiocyanate-phenol-chloroform (AGPC) protocol by the method mentioned above. The cDNA samples were synthesized by Improm-IITM Reverse Transcriptase kit (Promega Corporation, Madison, Wl) according to the manufacturer's instructions. Briefly, 5 ml of RNA and primer were added to 15 ml reverse transcription reaction mix (prepared by combining the following components of the Improm-IITM Reverse Transcriptase system). Annealing was performed by placing the tubes in a controlled-temperature heat block equilibrated at 25°C and incubated for 5 minutes. Extension was performed in a controlled-temperature heat block at 42°C for up to one hour. The extension temperature was optimized between 37°C and 55°C.

### Real-time PCR

Real time RT-PCR was performed in two laboratory sites where different PCR methodologies were used: the TaqMan^® ^Gold RT-PCR Kit without controls (Applied Biosystems, Foster City, CA), and the QuantiFast™ SYBR Green PCR kit (Qiagen, Hilden, Germany) according to the manufacturer's instructions. These two assays gave essentially the same results. For TaqMan^® ^assay, combinations of probes and primers of Table 5 were used. Real time PCR analysis was performed using an ABI Prism 7900 HT (Applied Biosystems) with the following thermal cycling conditions: 1 cycle at 50°C for 2 min, 1 cycle at 95°C for 10 min, followed by 40 cycles at 95°C for 15 sec and 60°C for 1 min. All samples were run in triplicate. Data was normalized against Irf1. The cDNA templates were quantified by the QuantiFast™ SYBR Green PCR kit according to the manufacturer's instructions. The sequences of the primers are shown in Table 7. Real time PCR reactions were carried out with 50 ng of total cDNA. The cycle parameters used were initial activation step at 95°C 5 min, denaturing at 95°C for 10 seconds, combined annealing and extension at 60°C for 30 seconds. After amplification, samples were kept at 55°C for 1 minute and the temperature was raised gradually by 0.5°C in every 10 seconds to perform the melt-curve analysis. All procedures for real time PCR were performed on the iCycler iQTM Real-Time PCR Detection System (Bio-Rad Laboratories, Tokyo, Japan) using the software of this instrument. All measurements were performed in triplicate. The threshold cycles (Ct) were used to quantify the mRNA expression levels of samples with GAPDH normalization. Some portions of the RT-PCR data are represented as heat maps in Additional File 8.

**Table 7:** The primer sequences used for SYBR Green RT-PCR

Specificty	Primer pair	Product size (bp)
Stat 1	5'-CACGCTGCCTATGATGTCTC-3' 5'-ACGCTTGCTTTTCCGTATGT-3'	132

Stat2	5'-CTTGTTCTTGGGTGGAGCACC-3' 5'-TTGGTGTAGGGCTGCACTGAG-3'	67

Isgf3g	5'-CATAGTTGGCACATGTGAGACA-3' 5'-ATCTCTCCAGCCGCTCTTAG-3'	121

Irf7	5'-CCCATCTTCGACTTCAGCAC-3' 5'-TGTAGTGTGGTGACCCTTGC-3'	88

Ifn-α	5'-GAATGCAACCCTCCTAGAC-3' 5'-GTCAGAGGAGGTTCCTG-3'	105

### Induction and quantitation of IFN-α protein

The frozen tissue was weighed, transferred to different tubes on ice, and homogenized in the proportion of 100 mg of the frozen tissue to 50 ml of PBS containing Complete Mini Protease Inhibitor Cocktail tablets (Roche Diagnostics; Indianapolis, IN) at a proportion of 1 tablet/10 ml at 4°C. The large intestine homogenates were centrifuged at 55 g for 30 seconds at 4°C through the gauze filter. The supernatant was then centrifuged at 2000 g for 3 minutes at 4°C. Supernatants were transferred to 5.0 mm filter tubes (MILLIPORE Corporation; Bedford, MA), centrifuged at 2000 g for 3 minutes at 4°C. IFN-α protein concentrations in the large intestine tissue homogenates were determined using an IFN-α Mouse ELISA kit (PBL Biomedical Laboratories; Piscataway NJ). Undiluted large intestine tissue homogenates were applied in duplicate to the ELISA plate.

### Statistical analyses

All data are expressed as the mean value ± S.E. Differences among groups were analyzed by Student's t-test and Fisher's least significant test, and p < 0.05 was considered significant for RT-PCR and ELISA experiments. Statistics for microarray analysis are described in the relevant *Methods *section and *Results *section.

## List of Abbreviations

JTX: juzentaihoto; ABMP: 2-Amino-5-bromo-6-methyl-4-pyrimidinol; IFN: type 1 interferon; SPF: specific pathogen-free; GF: Germ-free.

## Authors' contributions

KM participated in the design of the study, carried out the immunoassays and drafted the manuscript; KT, MN, NA, AM carried out the molecular genetic studies and performed the statistical analysis; KH, YO provided an animal; KW conceived of the study; MY participated in its design, coordination, performed the statistical analysis and helped to draft the manuscript. All authors read and approved the final manuscript.

## Supplementary Material

Additional file 1 **The upward effect of JTX on the gene expression in the large intestine in BALB/c SPF mice**. Click here for file

Additional file 2 **The downward effect of JTX on the gene expression in the large intestine in IQI SPF mice**. Click here for file

Additional file 3 **The upward effect of JTX on the gene expression in the small intestine in IQI SPF mice**. Click here for file

Additional file 4 **The downward effect of JTX on the gene expression in the small intestine in IQI SPF mice**. Click here for file

Additional file 5 **The upward effect of JTX on the gene expression in the large intestine in IQI GF mice**. Click here for file

Additional file 6 **Summary of altered gene lists**. Click here for file

Additional file 7 **The upward effect of JTX on the gene expression in the small intestine in IQI GF mice**. Click here for file

Additional file 8 **Heat maps for gene expression data**. (a) Gene chip data of all the probe sets whose MAS calls are "present" for all triplicate samples (a) and RT-PCR data of selected genes (b) were log-transformed and subjected to heat map generation. Click here for file
