# Supplementary material for: Microarray analysis on germfree mice elucidates the primary target of a traditional Japanese medicine juzentaihoto: acceleration of IFN-α response via affecting the ISGF3-IRF7 signaling cascade
Source: BMC Genomics. 2012 Jan 18;13:30. doi: 10.1186/1471-2164-13-30 (PMC3298487; doi:10.1186/1471-2164-13-30)
Supplement: Additional file 1 — The upward effect of JTX on the gene expression in the large intestine in BALB/c SPF mice. [file 1471-2164-13-30-S1.DOC]

Additional File 1. The upward effect of JTX on the gene expression in the large intestine in BALB/c SPF mice

| BALBLI-up |  |  |  |  |  |
| --- | --- | --- | --- | --- | --- |
| Probe Set ID | Gene Name | Gene Symbol | Entre ID | Fold Change | p-value |
| 102049_at | pyruvate dehydrogenase kinase, isoenzyme 4 | Pdk4 | 27273 | 2.51 | 0.002 |
| 98111_at | heat shock protein 110 | Hsp110 | 15505 | 2.06 | 0.061 |
| 97890_at | serum/glucocorticoid regulated kinase | Sgk | 20393 | 1.92 | 0.029 |
| 98994_at | solute carrier family 34 (sodium phosphate), member 2 | Slc34a2 | 20531 | 1.89 | 0.019 |
| 160901_at | FBJ osteosarcoma oncogene | Fos | 14281 | 1.86 | 0.066 |
| 102382_at | aryl hydrocarbon receptor nuclear translocator-like | Arntl | 11865 | 1.66 | 0.084 |
| 95465_s_at | transmembrane protein 37 | Tmem37 | 170706 | 1.62 | 0.006 |
| 160121_at | galactokinase 2 | Galk2 | 69976 | 1.59 | 0.056 |
| 95027_at | ubiquitin specific peptidase 21 | Usp21 | 30941 | 1.58 | 0.004 |
| 99535_at | CCR4 carbon catabolite repression 4-like (S. cerevisiae) | Ccrn4l | 12457 | 1.57 | 0.026 |
| 97684_at | protein interacting with C kinase 1 | Prkcabp | 18693 | 1.54 | 0.008 |
| 99972_at | tryptophan hydroxylase 1 | Tph1 | 21990 | 1.52 | 0.021 |
| 96204_at | SH3-binding domain glutamic acid-rich protein | Sh3bgr | 50795 | 1.51 | 0.022 |

The genes whose change was > 1.50 fold (BALBLI-up), with p < 0.1 (n=3, Welch's t test) were the listed sorted by fold-change. Unidentified 3 probe sets were omitted from the list.
