# Supplementary material for: Microarray analysis on germfree mice elucidates the primary target of a traditional Japanese medicine juzentaihoto: acceleration of IFN-α response via affecting the ISGF3-IRF7 signaling cascade
Source: BMC Genomics. 2012 Jan 18;13:30. doi: 10.1186/1471-2164-13-30 (PMC3298487; doi:10.1186/1471-2164-13-30)
Supplement: Additional file 2 — The downward effect of JTX on the gene expression in the large intestine in IQI SPF mice. [file 1471-2164-13-30-S2.DOC]

Additional File 2. The downward effect of JTX on the gene expression in the large intestine in IQI SPF mice

| SPFLI-down |  |  |  |  |  |
| --- | --- | --- | --- | --- | --- |
| Probe Set ID | Gene Name | Gene Symbol | Entre ID | Fold Change | p-value |
| 99551_f_at | defensin related cryptdin 5 | Defcr5 | 13239 | 0.44 | 0.066 |
| 102239_at | B-cell leukemia/lymphoma 3 | Bcl3 | 12051 | 0.47 | 0.076 |
| 96094_at | apolipoprotein A-I | Apoa1 | 11806 | 0.51 | 0.073 |
| 160894_at | CCAAT/enhancer binding protein (C/EBP), delta | Cebpd | 12609 | 0.54 | 0.006 |
| 160901_at | FBJ osteosarcoma oncogene | Fos | 14281 | 0.54 | 0.025 |
| 99603_g_at | Kruppel-like factor 10 | Klf10 | 21847 | 0.56 | 0.009 |
| 93342_at | Mki67 (FHA domain) interacting nucleolar phosphoprotein | Mki67ip | 67949 | 0.57 | 0.076 |
| 102850_at | tyrosine kinase, non-receptor, 2 | Tnk2 | 51789 | 0.59 | 0.020 |
| 94781_at | hemoglobin alpha, adult chain 1 | Hba-a1 | 15122 | 0.61 | 0.033 |
| 103499_at | Von Willebrand factor homolog | Vwf | 22371 | 0.61 | 0.080 |
| 92830_s_at | zinc finger protein 36 | Zfp36 | 22695 | 0.62 | 0.040 |
| 93367_at | telomerase associated protein 1 | Tep1 | 21745 | 0.64 | 0.098 |
| 100910_at | surfeit gene 2 | Surf2 | 20931 | 0.64 | 0.051 |
| 95665_at | SEC14-like 1 (S. cerevisiae) | Sec14l1 | 74136 | 0.66 | 0.081 |
| 98612_at | cytochrome P450, family 2, subfamily d, polypeptide 10 | Cyp2d10 | 13101 | 0.66 | 0.084 |
| 95393_at | BTB (POZ) domain containing 3 | Btbd3 | 228662 | 0.67 | 0.057 |

The genes whose change was < 0.67 fold with p < 0.1 (n=3, Welch's t test) were the listed sorted by fold-change. Unidentified 4 probe sets were omitted from the list.
