# Supplementary material for: Microarray analysis on germfree mice elucidates the primary target of a traditional Japanese medicine juzentaihoto: acceleration of IFN-α response via affecting the ISGF3-IRF7 signaling cascade
Source: BMC Genomics. 2012 Jan 18;13:30. doi: 10.1186/1471-2164-13-30 (PMC3298487; doi:10.1186/1471-2164-13-30)
Supplement: Additional file 3 — The upward effect of JTX on the gene expression in the small intestine in IQI SPF mice. [file 1471-2164-13-30-S3.DOC]

Additional File 3. The upward effect of JTX on the gene expression in the small intestine in IQI SPF mice

| SPFSI-up |  | | |  |  |  |  |
| --- | --- | --- | --- | --- | --- | --- | --- |
| Probe Set ID | Gene Name | | | Gene Symbol | Entre ID | Fold Change | p-value |
| 99076_at | nuclear receptor subfamily 1, group D, member 2 | | | Nr1d2 | 353187 | 4.98 | 0.057 |
| 102049_at | pyruvate dehydrogenase kinase, isoenzyme 4 | | | Pdk4 | 27273 | 3.07 | 0.051 |
| 103983_at | alcohol dehydrogenase 4 (class II), pi polypeptide | | | Adh4 | 26876 | 3.05 | 0.062 |
| 99549_at | Osteoglycin | | | Ogn | 18295 | 2.81 | 0.018 |
| 92454_at | solute carrier family 6 (neurotransmitter transporter), member 20 | | | Slc6a20 | 22599 | 2.60 | 0.003 |
| 103957_at | transferrin receptor | | | Tfrc | 22042 | 2.43 | 0.005 |
| 94238_at | protease, serine, 23 | | | Prss23 | 76453 | 2.38 | 0.053 |
| 94057_g_at | stearoyl-Coenzyme A desaturase 1 | | | Scd1 | 20249 | 2.36 | 0.000 |
| 100494_at | fibroblast growth factor 1 | | | Fgf1 | 14164 | 2.34 | 0.045 |
| 103982_s_at | alcohol dehydrogenase 4 (class II), pi polypeptide | | | Adh4 | 26876 | 2.33 | 0.067 |
| 104412_at | guanine nucleotide binding protein, alpha inhibiting 1 | | | Gnai1 | 14677 | 2.25 | 0.039 |
| 160280_at | caveolin, caveolae protein 1 | | | Cav1 | 12389 | 2.24 | 0.041 |
| 99642_i_at | carboxypeptidase E /// similar to carboxypeptidase E | | | Cpe | 12876 | 2.21 | 0.093 |
| 93411_at | sema domain, immunoglobulin domain (Ig), and GPI membrane anchor, (semaphorin) 7A | | | Sema7a | 20361 | 2.16 | 0.014 |
| 160464_s_at | N-myc downstream regulated-like | | | Ndrl | 17990 | 2.15 | 0.031 |
| 100112_at | chemokine (C-X-C motif) ligand 12 | | | Cxcl12 | 20315 | 2.14 | 0.005 |
| 92593_at | periostin, osteoblast specific factor | | | Postn | 50706 | 2.11 | 0.031 |
| 97549_at | cofilin 2, muscle | | | Cfl2 | 12632 | 2.10 | 0.035 |
| 93330_at | aquaporin 1 | | | Aqp1 | 11826 | 2.10 | 0.009 |
| 96946_at | LSM1 homolog, U6 small nuclear RNA associated (S. cerevisiae) | | | Lsm1 | 67207 | 2.05 | 0.079 |
| 94906_at | alcohol dehydrogenase 1 (class I) | | | Adh1 | 11522 | 2.03 | 0.065 |
| 103050_at | Transcription factor 21 | | | Tcf21 | 21412 | 2.01 | 0.011 |
| 95000_g_at | cubilin (intrinsic factor-cobalamin receptor) | | | Cubn | 65969 | 1.97 | 0.063 |
| 95379_at | mab-21-like 2 (C. elegans) | | | Mab21l2 | 23937 | 1.97 | 0.091 |
| 101422_at | formin binding protein 4 | | | Fnbp4 | 55935 | 1.97 | 0.011 |
| 97500_g_at | four and a half LIM domains 1 | | | Fhl1 | 14199 | 1.95 | 0.064 |
| 101561_at | metallothionein 2 | | | Mt2 | 17750 | 1.93 | 0.031 |
| 100393_at | vasoactive intestinal polypeptide | | | Vip | 22353 | 1.92 | 0.003 |
| 160373_i_at | serum deprivation response | | | Sdpr | 20324 | 1.92 | 0.076 |
| 96796_f_at | UDP glucuronosyltransferase 2 family, polypeptide B5 | | | Ugt2b5 | 22238 | 1.90 | 0.076 |
| 94540_at | cytochrome P450, family 2, subfamily d, polypeptide 26 | | | Cyp2d26 | 76279 | 1.89 | 0.013 |
| 162428_i_at | S100 calcium binding protein A14 | | | S100a14 | 66166 | 1.89 | 0.080 |
| 96144_at | inhibitor of DNA binding 4 | | | Id4 | 15904 | 1.88 | 0.006 |
| 96026_at | S-adenosylhomocysteine hydrolase | | | Ahcy | 269378 | 1.88 | 0.025 |
| 93542_at | phosphotriesterase related | | | Pter | 19212 | 1.87 | 0.023 |
| 96774_at | pleckstrin homology domain containing, family C (with FERM domain) member 1 | | | Plekhc1 | 218952 | 1.83 | 0.013 |
| 104618_at | retinoblastoma binding protein 9 | | | Rbbp9 | 26450 | 1.83 | 0.090 |
| 102123_at | Lysosomal acid lipase 1 | | | Lip1 | 16889 | 1.83 | 0.001 |
| 95052_at | C1q domain containing 2 | | | C1qdc2 | 67389 | 1.83 | 0.063 |
| 100634_at | retinol dehydrogenase 7 | | | Rdh7 | 54150 | 1.83 | 0.039 |
| 93353_at | Lumican | | | Lum | 17022 | 1.82 | 0.007 |
| 97297_at | Purkinje cell protein 4-like 1 | | | Pcp4l1 | 66425 | 1.81 | 0.007 |
| 93056_g_at | ankyrin repeat domain 46 | | | Ankrd46 | 68839 | 1.80 | 0.057 |
| 98979_at | menage a trois 1 | | | Mnat1 | 17420 | 1.80 | 0.027 |
| 92857_at | ribosomal protein L22 | | | Rpl22 | 19934 | 1.80 | 0.056 |
| 95670_at | stathmin-like 2 | | | Stmn2 | 20257 | 1.79 | 0.086 |
| 160667_at | Ena-vasodilator stimulated phosphoprotein | | | Evl | 14026 | 1.78 | 0.047 |
| 101089_at | PDZ and LIM domain 3 | | | Pdlim3 | 53318 | 1.78 | 0.018 |
| 92738_at | glial cell line derived neurotrophic factor | | | Gdnf | 14573 | 1.77 | 0.084 |
| 96957_at | calcium and integrin binding 1 (calmyrin) | | | Cib1 | 23991 | 1.77 | 0.065 |
| 93284_at | cold inducible RNA binding protein | | | Cirbp | 12696 | 1.77 | 0.078 |
| 102225_at | RAB GTPase activating protein 1-like | | | Rabgap1l | 29809 | 1.76 | 0.086 |
| 97406_at | core-binding factor, runt domain, alpha subunit 2, translocated to, 2 homolog (human) | | | Cbfa2t2h | 12396 | 1.75 | 0.002 |
| 104327_at | expressed sequence AI265322 | | | AI265322 | 106543 | 1.75 | 0.094 |
| 95104_at | syndecan 2 | | | Sdc2 | 15529 | 1.75 | 0.004 |
| 97430_at | solute carrier family 37 (glycerol-6-phosphate transporter), member 4 | | | Slc37a4 | 14385 | 1.75 | 0.092 |
| 94524_at | death associated protein 3 | | | Dap3 | 65111 | 1.74 | 0.004 |
| 162340_r_at | S100 calcium binding protein A10 (calpactin) | | | S100a10 | 20194 | 1.73 | 0.043 |
| 101513_at | prefoldin 5 | | | *1 | | 1.73 | 0.089 |
| 99107_at | growth hormone receptor | | | Ghr | 14600 | 1.73 | 0.076 |
| 95016_at | neuropilin 1 | | | Nrp1 | 18186 | 1.72 | 0.035 |
| 99875_at | Hairless | | | Hr | 15460 | 1.71 | 0.069 |
| 95531_at | Angiomotin | | | Amot | 27494 | 1.71 | 0.018 |
| 103809_r_at | dynein cytoplasmic 1 intermediate chain 1 | | | Dncic1 | 13426 | 1.71 | 0.035 |
| 104031_at | patched homolog 1 | | | Ptch1 | 19206 | 1.71 | 0.066 |
| 101034_at | growth factor receptor bound protein 2 | | | Grb2 | 14784 | 1.69 | 0.035 |
| 160669_at | HIV TAT specific factor 1 | | | Htatsf1 | 72459 | 1.69 | 0.004 |
| 98946_at | WD repeat and SOCS box-containing 1 | | | Wsb1 | 78889 | 1.69 | 0.054 |
| 99643_f_at | carboxypeptidase E /// similar to carboxypeptidase E | | | Cpe | 12876 | 1.69 | 0.065 |
| 97797_at | asparagine synthetase domain containing 1 | | | *2 | | 1.68 | 0.040 |
| 101966_s_at | ring finger protein 13 | | | Rnf13 | 24017 | 1.68 | 0.092 |
| 92477_at | Spindling | | | Spin | 20729 | 1.67 | 0.054 |
| 99014_at | amyloid beta (A4) precursor protein-binding, family B, member 1 | | | Apbb1 | 11785 | 1.66 | 0.034 |
| 160537_at | sulfotransferase family 1D, member 1 | | | Sult1d1 | 53315 | 1.66 | 0.096 |
| 104535_at | YME1-like 1 (S. cerevisiae) | | | Yme1l1 | 27377 | 1.65 | 0.034 |
| 101469_at | neural precursor cell expressed, developmentally down-regulated gene 9 | | | Nedd9 | 18003 | 1.65 | 0.026 |
| 93378_at | homeo box C8 | | | Hoxc8 | 15426 | 1.63 | 0.055 |
| 97515_at | hydroxysteroid (17-beta) dehydrogenase 4 | | | Hsd17b4 | 15488 | 1.63 | 0.020 |
| 104550_at | cytochrome P450, family 2, subfamily s, polypeptide 1 | | | Cyp2s1 | 74134 | 1.63 | 0.047 |
| 103991_at | alkaline phosphatase 5 | | | Akp5 | 11650 | 1.63 | 0.030 |
| 160319_at | SPARC-like 1 (mast9, hevin) | | | Sparcl1 | 13602 | 1.62 | 0.087 |
| 160923_at | amiloride binding protein 1 (amine oxidase, copper-containing) | | | Abp1 | 76507 | 1.62 | 0.060 |
| 98782_at | complexin 2 | | | Cplx2 | 12890 | 1.62 | 0.012 |
| 102235_at | v-myc myelocytomatosis viral oncogene homolog 1, lung carcinoma derived (avian) | | | Lmyc1 | 16918 | 1.62 | 0.014 |
| 95044_at | mediator of RNA polymerase II transcription, subunit 28 homolog (yeast) | | | Med28 | 66999 | 1.61 | 0.009 |
| 92185_at | ADP-ribosylation factor-like 4C | | | Arl7 | 320982 | 1.61 | 0.024 |
| 160519_at | tissue inhibitor of metalloproteinase 3 | | | Timp3 | 21859 | 1.61 | 0.041 |
| 103100_at | WW domain containing transcription regulator 1 | | | Wwtr1 | 97064 | 1.61 | 0.016 |
| 94964_at | Vinculin | | | Vcl | 22330 | 1.61 | 0.091 |
| 94394_at | Harvey rat sarcoma oncogene, subgroup R | | | Rras | 20130 | 1.61 | 0.084 |
| 103047_at | Peroxisomal membrane protein 3 | | | Pxmp3 | 19302 | 1.60 | 0.065 |
| 104728_at | protein S (alpha) | | | Pros1 | 19128 | 1.60 | 0.001 |
| 104322_at | Cytoskeleton associated protein 2 | | | Ckap2 | 80986 | 1.60 | 0.071 |
| 100916_at | solute carrier family 22 (organic cation transporter), member 1 | | | Slc22a1 | 20517 | 1.59 | 0.050 |
| 94706_s_at | eyes absent 1 homolog (Drosophila) | | | Eya1 | 14048 | 1.59 | 0.001 |
| 96351_at | basic transcription factor 3-like 4 | | | Btf3l4 | 70533 | 1.59 | 0.057 |
| 100307_at | nuclear factor I/X | | | Nfix | 18032 | 1.58 | 0.057 |
| 103715_at | Scinderin | | | Scin | 20259 | 1.58 | 0.071 |
| 93614_at | Ras-related GTP binding D | | | Rragd | 52187 | 1.57 | 0.063 |
| 93543_f_at | glutathione S-transferase, mu 1 | | | Gstm1 | 14862 | 1.57 | 0.079 |
| 98976_at | ADAM-like, decysin 1 | | | Adamdec1 | 58860 | 1.57 | 0.098 |
| 94555_at | ring finger protein 167 | | | Rnf167 | 70510 | 1.56 | 0.087 |
| 96764_at | interferon inducible GTPase 1 | | | Iigp1 | 60440 | 1.56 | 0.007 |
| 160827_at | Ras and Rab interactor 2 | | | *3 | | 1.56 | 0.018 |
| 93731_at | FK506 binding protein 9 | | | Fkbp9 | 27055 | 1.55 | 0.057 |
| 94359_at | pleckstrin homology domain containing, family A (phosphoinositide binding specific) member 1 | | | Plekha1 | 101476 | 1.55 | 0.003 |
| 101949_at | phosphomannomutase 2 | | | Pmm2 | 54128 | 1.55 | 0.074 |
| 101861_at | sarcoglycan, epsilon | | | Sgce | 20392 | 1.54 | 0.059 |
| 160085_at | thiosulfate sulfurtransferase, mitochondrial | | | Tst | 22117 | 1.54 | 0.042 |
| 93320_at | carnitine palmitoyltransferase 1a, liver | | | Cpt1a | 12894 | 1.54 | 0.046 |
| 94545_at | reticulon 1 | | | Rtn1 | 104001 | 1.54 | 0.045 |
| 99559_at | aldehyde dehydrogenase family 3, subfamily A2 | | | Aldh3a2 | 11671 | 1.53 | 0.099 |
| 92608_at | cysteine and glycine-rich protein 1 | | | Csrp1 | 13007 | 1.53 | 0.050 |
| 95883_at | PHD finger protein 17 | | | Phf17 | 269424 | 1.53 | 0.094 |
| 160625_f_at | methyltransferase like 8 | | | BC004636 | 228019 | 1.53 | 0.049 |
| 99637_at | procollagen, type XV  WD repeat domain 26 | | | Col15a1 | 12819 | 1.53 | 0.097 |
| 104612_g_at |  |  |  | Wdr26 | 226757 | 1.53 | 0.092 |
| 103812_at | chloride channel calcium activated 1 | | | Clca1 | 12722 | 1.53 | 0.037 |
| 160188_at | nudix (nucleoside diphosphate linked moiety X)-type motif 4 | | | Nudt4 | 71207 | 1.52 | 0.008 |
| 95701_at | CXXC finger 5 | | | Cxxc5 | 67393 | 1.52 | 0.088 |
| 161826_r_at | glutamate-ammonia ligase (glutamine synthetase) | | | Glul | 14645 | 1.51 | 0.011 |
| 100941_at | protein inhibitor of activated STAT 2 | | | Pias2 | 17344  16535 | 1.51 | 0.001 |
| 99322_at | potassium voltage-gated channel, subfamily Q, member 1 | | | Kcnq1 |  | 1.50 | 0.087 |
| 104706_at | peroxisome biogenesis factor 7 | | | Pex7 | 18634 | 1.50 | 0.037 |
| 96255_at | BCL2/adenovirus E1B interacting protein 3-like | | | Bnip3l | 12177 | 1.50 | 0.100 |
| 95483_at | | proteasome (prosome, macropain) 26S subunit, non-ATPase, 1 | Psmd1 | | 70247 | 0.67 | 0.031 |

The genes whose change > 1.50 fold with p < 0.1 (n=3, Welch's t test) were the listed sorted byfold-change. Unidentified 16 probe sets were omitted from the list. *1 Gene Symbol, Entre ID : Pfdn5 /// LOC434860, 434860 /// 56612. *2 Gene Symbol, Entre ID : 2210409 M. *3 Gene Symbol, Entre ID : Rin2 /// LOC547368, 547368 /// 74030
