# Supplementary material for: Microarray analysis on germfree mice elucidates the primary target of a traditional Japanese medicine juzentaihoto: acceleration of IFN-α response via affecting the ISGF3-IRF7 signaling cascade
Source: BMC Genomics. 2012 Jan 18;13:30. doi: 10.1186/1471-2164-13-30 (PMC3298487; doi:10.1186/1471-2164-13-30)
Supplement: Additional file 4 — The downward effect of JTX on the gene expression in the small intestine in IQI SPF mice. [file 1471-2164-13-30-S4.DOC]

Additional File 4. The downward effect of JTX on the gene expression in the small intestine in IQI SPF mice

| SPFSI-down |  |  |  |  |  |
| --- | --- | --- | --- | --- | --- |
| Probe Set ID | Gene Name | Gene Symbol | Entre ID | Fold Change | p-value |
| 94728_f_at | mast cell protease 1 | Mcpt1 | 17224 | 0.28 | 0.045 |
| 102823_at | Immunoglobulin heavy chain (gamma polypeptide) | Ighg | 380794 | 0.33 | 0.054 |
| 160894_at | CCAAT/enhancer binding protein (C/EBP), delta | Cebpd | 12609 | 0.42 | 0.010 |
| 97098_at | threonine aldolase 1 | Tha1 | 71776 | 0.43 | 0.023 |
| 98111_at | heat shock protein 110 | Hsp110 | 15505 | 0.44 | 0.021 |
| 92735_at | phospholipase A2, group IIA (platelets, synovial fluid) | Pla2g2a | 18780 | 0.48 | 0.080 |
| 162041_f_at | mucin 13, epithelial transmembrane | Muc13 | 17063 | 0.50 | 0.088 |
| 97269_f_at | keratinocyte associated protein 2 | Krtcap2 | 66059 | 0.55 | 0.036 |
| 102415_r_at | DnaJ (Hsp40) homolog, subfamily C, member 3 | Dnajc3 | 19107 | 0.57 | 0.068 |
| 92689_at | interleukin 18 binding protein | Il18bp | 16068 | 0.58 | 0.093 |
| 94420_f_at | cryptochrome 1 (photolyase-like) | Cry1 | 12952 | 0.60 | 0.017 |
| 104134_at | ganglioside-induced differentiation-associated-protein 2 | Gdap2 | 14547 | 0.61 | 0.079 |
| 97719_at | macrophage stimulating 1 receptor (c-met-related tyrosine kinase) | Mst1r | 19882 | 0.61 | 0.098 |
| 102239_at | B-cell leukemia/lymphoma 3 | Bcl3 | 12051 | 0.61 | 0.076 |
| 98547_at | mitochondrial ribosomal protein S12 | Mrps12 | 24030 | 0.61 | 0.032 |
| 160737_at | lanosterol synthase | Lss | 16987 | 0.64 | 0.100 |
| 97551_at | huntingtin interacting protein 1 related | Hip1r | 29816 | 0.64 | 0.065 |
| 96123_at | lipopolysaccharide binding protein | Lbp | 16803 | 0.64 | 0.098 |
| 161013_f_at | splA/ryanodine receptor domain and SOCS box containing 1 | Spsb1 | 74646 | 0.64 | 0.038 |
| 160770_at | mevalonate (diphospho) decarboxylase | Mvd | 192156 | 0.65 | 0.062 |
| 161817_f_at | splA/ryanodine receptor domain and SOCS box containing 1 | Spsb1 | 74646 | 0.65 | 0.073 |
| 96298_f_at | dynein light chain LC8-type 1 | Dnclc1 | 56455 | 0.65 | 0.051 |
| 103862_r_at | DNA segment, Chr 7, Wayne State University 128, expressed | D7Wsu128e | 28018 | 0.65 | 0.009 |
| 99982_at | nuclear factor of kappa light chain gene enhancer in B-cells inhibitor, beta | Nfkbib | 18036 | 0.66 | 0.052 |
| 101426_at | ceramide kinase | Cerk | 223753 | 0.67 | 0.065 |
| 92636_f_at | SEC61, gamma subunit | Sec61g | 20335 | 0.67 | 0.012 |
| 94918_at | alanyl-tRNA synthetase | Aars | 234734 | 0.67 | 0.044 |
| 95483_at | proteasome (prosome, macropain) 26S subunit, non-ATPase, 1 | Psmd1 | 70247 | 0.67 | 0.031 |

The genes whose change < 0.67 fold with p < 0.1 (n=3, Welch's t test) were the listed sorted by fold-change. Unidentified 1 probe set was omitted from the list.
