# Supplementary material for: Microarray analysis on germfree mice elucidates the primary target of a traditional Japanese medicine juzentaihoto: acceleration of IFN-α response via affecting the ISGF3-IRF7 signaling cascade
Source: BMC Genomics. 2012 Jan 18;13:30. doi: 10.1186/1471-2164-13-30 (PMC3298487; doi:10.1186/1471-2164-13-30)
Supplement: Additional file 5 — The upward effect of JTX on the gene expression in the large intestine in IQI GF mice. [file 1471-2164-13-30-S5.DOC]

Additional File 5. The upward effect of JTX on the gene expression in the large intestine in IQI GFF mice

|  | GFLI-up | |  | | |  | |  | |  | |  |
| --- | --- | --- | --- | --- | --- | --- | --- | --- | --- | --- | --- | --- |
|  | Probe Set ID | | Gene Name | | | Gene Symbol | | Entre ID | | Fold Change | | p-value |
| 99126_at | | inactive X specific transcripts | | Xist | 213742 | | 138.98 | | 0.010 | |  | |
| 103534_at | | hemoglobin, beta adult minor chain | | Hbb-b2 | 15130 | | 1.86 | | 0.060 | |  | |
| 101979_at | | growth arrest and DNA-damage-inducible 45 gamma | | Gadd45g | 23882 | | 1.80 | | 0.020 | |  | |
| 161907_s_at | | tenascin XB | | Tnxb | 81877 | | 1.75 | | 0.004 | |  | |
| 94781_at | | hemoglobin alpha, adult chain 1 | | Hba-a1 | 15122 | | 1.73 | | 0.063 | |  | |
| 99658_f_at | | fumarylacetoacetate hydrolase domain containing 1 | | Fahd1 | 68636 | | 1.71 | | 0.013 | |  | |
| 93975_at | | ERBB receptor feedback inhibitor 1 | | Errfi1 | 74155 | | 1.69 | | 0.040 | |  | |
| 93636_at | | Rotatin | | Rttn | 246102 | | 1.67 | | 0.039 | |  | |
| 101295_s_at | | chloride channel, nucleotide-sensitive, 1A | | Clns1a | 12729 | | 1.65 | | 0.081 | |  | |
| 93309_at | | DEAD/H (Asp-Glu-Ala-Asp/His) box polypeptide 3, X-linked | | Ddx3x | 13205 | | 1.62 | | 0.031 | |  | |
| 102300_at | | receptor-associated protein of the synapse | | Rapsn | 19400 | | 1.59 | | 0.067 | |  | |
| 161085_r_at | | alpha 1,4-galactosyltransferase | | A4galt | 239559 | | 1.58 | | 0.015 | |  | |
| 96539_at | | serpine1 mRNA binding protein 1 | | Serbp1 | 66870 | | 1.56 | | 0.001 | |  | |
| 98834_at | | integrin alpha 2 | | Itga2 | 16398 | | 1.55 | | 0.018 | |  | |
| 97529_at | | annexin A8 | | Anxa8 | 11752 | | 1.54 | | 0.039 | |  | |
| 92745_at | | homeobox A9 | | Hoxa9 | 15405 | | 1.51 | | 0.006 | |  | |

The genes whose change > 1.50 fold with p < 0.1 (n=3, Welch's t test) were the listed sorted by fold-change. Unidentified 2 probe sets were omitted from the list.
