# Supplementary material for: Microarray analysis on germfree mice elucidates the primary target of a traditional Japanese medicine juzentaihoto: acceleration of IFN-α response via affecting the ISGF3-IRF7 signaling cascade
Source: BMC Genomics. 2012 Jan 18;13:30. doi: 10.1186/1471-2164-13-30 (PMC3298487; doi:10.1186/1471-2164-13-30)
Supplement: Additional file 6 — Summary of altered gene lists. [file 1471-2164-13-30-S6.DOC]

Additional File 6. Summary of altered gene lists

| **Strain** | Status | Organ | Change | Table number |
| --- | --- | --- | --- | --- |
| IQI | SPF | Large intestine | Up | Table 1 |
|  |  |  | Down | Additional File 2 |
|  |  | Small intestine | Up | Additional File 3 |
|  |  |  | Down | Additional File 4 |
|  | GF | Large intestine | Up | Additional File 5 |
|  |  |  | Down | Table 3 |
|  |  | Small intestine | Up | Additional File 7 |
|  |  |  | Down | Table 4 |
| Balb/c | SPF | Large intestine | Up | Additional File 1 |
|  |  |  | Down | Table 2 |
