# Supplementary material for: Microarray analysis on germfree mice elucidates the primary target of a traditional Japanese medicine juzentaihoto: acceleration of IFN-α response via affecting the ISGF3-IRF7 signaling cascade
Source: BMC Genomics. 2012 Jan 18;13:30. doi: 10.1186/1471-2164-13-30 (PMC3298487; doi:10.1186/1471-2164-13-30)
Supplement: Additional file 7 — The upward effect of JTX on the gene expression in the small intestine in IQI GF mice. [file 1471-2164-13-30-S7.DOC]

Additional File 7. The upward effect of JTX on the gene expression in the small intestine in IQI GF mice

| GFSI-up |  |  |  |  |  |
| --- | --- | --- | --- | --- | --- |
| Probe Set ID | Gene Name | Gene Symbol | Entre ID | Fold Change | p-value |
| 99126_at | inactive X specific transcripts | Xist | 213742 | 103.64 | 0.030 |
| 162428_i_at | S100 calcium binding protein A14 | S100a14 | 66166 | 2.52 | 0.000 |
| 101290_at | gonadotropin releasing hormone 1 | Gnrh1 | 14714 | 1.86 | 0.037 |
| 102986_at | myogenic differentiation 1 | Myod1 | 17927 | 1.86 | 0.014 |
| 97472_at | solute carrier family 25 (mitochondrial carrier, peroxisomal membrane protein), member 17 | Slc25a17 | 20524 | 1.82 | 0.022 |
| 92352_at | endothelial differentiation, sphingolipid G-protein-coupled receptor, 3 | Edg3 | 13610 | 1.79 | 0.030 |
| 103921_i_at | cytochrome b5 reductase 1 | Cyb5r1 | 72017 | 1.78 | 0.024 |
| 161432_f_at | squamous cell carcinoma antigen recognized by T-cells 3 | Sart3 | 53890 | 1.76 | 0.000 |
| 99476_at | procollagen, type XIV, alpha 1 | Col14a1 | 12818 | 1.73 | 0.009 |
| 161907_s_at | tenascin XB | Tnxb | 81877 | 1.70 | 0.040 |
| 98817_at | Follistatin | Fst | 14313 | 1.69 | 0.008 |
| 93836_at | BCL2/adenovirus E1B interacting protein 1, NIP3 | Bnip3 | 12176 | 1.69 | 0.008 |
| 99856_r_at | catenin (cadherin associated protein), delta 2 | Ctnnd2 | 18163 | 1.66 | 0.043 |
| 103420_at | Emerin | Emd | 13726 | 1.64 | 0.013 |
| 161894_r_at | splicing factor 1 | Sf1 | 22668 | 1.62 | 0.037 |
| 101458_at | wee 1 homolog (S. pombe) | Wee1 | 22390 | 1.62 | 0.012 |
| 92838_at | fascin homolog 1, actin bundling protein (Strongylocentrotus) purpuratus) | Fscn1 | 14086 | 1.62 | 0.039 |
| 161304_r_at | guanine nucleotide binding protein, related sequence 1 | Gna-rs1 | 14670 | 1.61 | 0.001 |
| 103037_at | cardiotrophin 1 | Ctf1 | 13019 | 1.60 | 0.002 |
| 98800_at | solute carrier family 23 (nucleobase transporters), member 3 | Slc23a3 | 22626 | 1.60 | 0.023 |
| 160559_at | proteasome (prosome, macropain) subunit, beta type 5 | Psmb5 | 19173 | 1.60 | 0.001 |
| 104598_at | dual specificity phosphatase 1 | Dusp1 | 19252 | 1.59 | 0.045 |
| 93866_s_at | matrix Gla protein | Mgp | 17313 | 1.58 | 0.040 |
| 96939_at | myosin, light polypeptide 9, regulatory | Myl9 | 98932 | 1.58 | 0.033 |
| 161888_r_at | COMM domain containing 9 | Commd9 | 76501 | 1.57 | 0.007 |
| 94373_at | dolichyl pyrophosphate phosphatase 1 | Dolpp1 | 57170 | 1.56 | 0.009 |
| 161071_at | glutaminyl-tRNA synthase (glutamine-hydrolyzing)-like 1 | Qrsl1 | 76563 | 1.54 | 0.035 |
| 101735_f_at | angiogenin, ribonuclease A family, member 2 | Ang2 | 11731 | 1.54 | 0.016 |
| 103294_at | regulator of G-protein signaling 5 | Rgs5 | 19737 | 1.53 | 0.036 |
| 161112_at | poly (ADP-ribose) polymerase family, member 16 | Parp16 | 214424 | 1.52 | 0.035 |
| 160128_at | propionyl Coenzyme A carboxylase, beta polypeptide | Pccb | 66904 | 1.52 | 0.018 |
| 94304_at | annexin A6 | Anxa6 | 11749 | 1.52 | 0.026 |
| 99866_at | RNA binding motif, single stranded interacting protein 1 | Rbms1 | 56878 | 1.52 | 0.022 |
| 160192_at | RNA binding motif protein, X chromosome retrogene | Rbmxrt | 19656 | 1.51 | 0.013 |

The genes whose change was > 1.50 fold, with p < 0.1 (n=3, Welch's t test) were the listed sorted by fold-change. Seven unidentified probe sets were omitted from the list.
