# Supplementary material for: Microarray analysis on germfree mice elucidates the primary target of a traditional Japanese medicine juzentaihoto: acceleration of IFN-α response via affecting the ISGF3-IRF7 signaling cascade
Source: BMC Genomics. 2012 Jan 18;13:30. doi: 10.1186/1471-2164-13-30 (PMC3298487; doi:10.1186/1471-2164-13-30)
Supplement: Additional file 8 — Heat maps for gene expression data. (a) Gene chip data of all the probe sets whose MAS calls are "present" for all triplicate samples (a) and RT-PCR data of selected genes (b) were log-transformed and subjected to heat map generation. [file 1471-2164-13-30-S8.DOC]

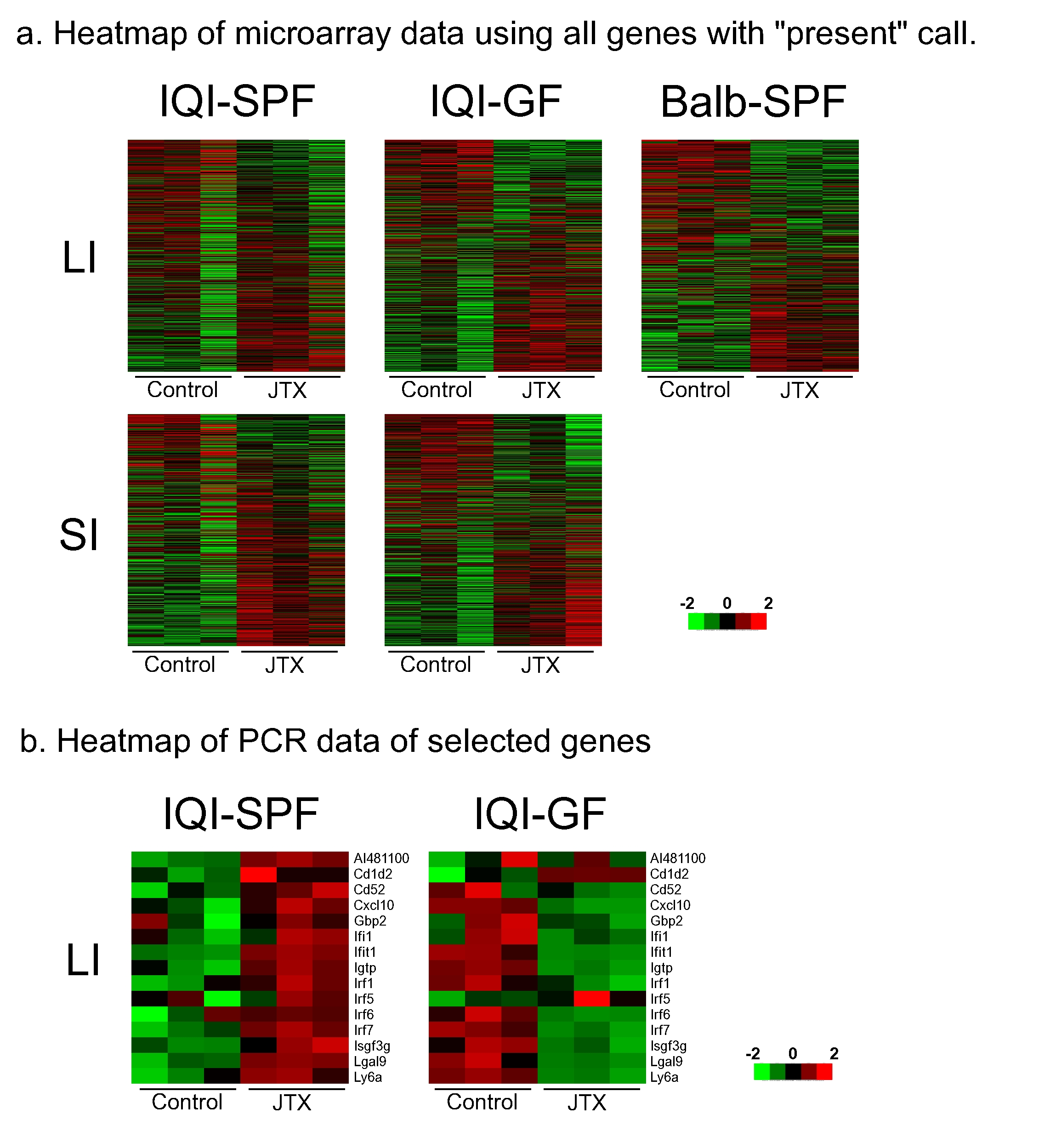


**Additional File 8.** Heat maps for gene expression data. (a) Gene chip data of all the probe sets whose MAS calls are “present” for all triplicate samples (a) and RT-PCR data of selected genes (b) were log-transformed and subjected to heat map generation. Heat maps were generated using the R statistical computing environment (<http://www.r-project.org/>). LI: large intestine, SI: small intestine
